# Endocytosis mediated by an atypical CUBAM complex modulates slit diaphragm dynamics in nephrocytes

**DOI:** 10.1242/dev.199894

**Published:** 2021-11-30

**Authors:** Alexandra Atienza-Manuel, Vicente Castillo-Mancho, Stefano De Renzis, Joaquim Culi, Mar Ruiz-Gómez

**Affiliations:** 1 Centro de Biología Molecular Severo Ochoa, CSIC and UAM, Nicolás Cabrera 1, Cantoblanco 28049, Madrid, Spain; 2 European Molecular Biology Laboratory (EMBL), Meyerhofstrasse 1, 69117 Heidelberg, Germany

**Keywords:** CUBAM endocytic receptor, Slit diaphragm, Nephrocyte, *Drosophila*

## Abstract

The vertebrate endocytic receptor CUBAM, consisting of three cubilin monomers complexed with a single amnionless molecule, plays a major role in protein reabsorption in the renal proximal tubule. Here, we show that *Drosophila* CUBAM is a tripartite complex composed of Amnionless and two cubilin paralogues, Cubilin and Cubilin2, and that it is required for nephrocyte slit diaphragm (SD) dynamics. Loss of CUBAM-mediated endocytosis induces dramatic morphological changes in nephrocytes and promotes enlarged ingressions of the external membrane and SD mislocalisation. These phenotypes result in part from an imbalance between endocytosis, which is strongly impaired in CUBAM mutants, and exocytosis in these highly active cells. Of note, rescuing receptor-mediated endocytosis by Megalin/LRP2 or Rab5 expression only partially restores SD positioning in CUBAM mutants, suggesting a specific requirement of CUBAM in SD degradation and/or recycling. This finding and the reported expression of CUBAM in podocytes suggest a possible unexpected conserved role for this endocytic receptor in vertebrate SD remodelling.

## Abstract (precis)

**Summary:** A genetic study revealing that endocytosis mediated by an atypical CUBAM endocytic receptor, composed of Amnionless and two Cubilin paralogues, regulates slit diaphragm remodelling in *Drosophila* nephrocytes.

## INTRODUCTION

The seminal discovery that *Drosophila* nephrocytes, the excretory cells engaged in haemolymph ultrafiltration, had filtration diaphragms analogous to the slit diaphragm (SD) of vertebrate podocytes (Weavers et al., 2009; Zhuang et al., 2009) opened the door to many studies that used them to examine different aspects of SD formation and stability (Carrasco-Rando et al., 2019; Tutor et al., 2014). Moreover, *Drosophila* nephrocytes also helped to model nephropathies (Hermle et al., 2016; Na et al., 2015) and to investigate the effects on podocyte function of specific mutations associated with steroid-resistant nephrotic syndromes by generating the corresponding fly avatars (Gonçalves et al., 2018; Lovric et al., 2017; Zhao et al., 2019; Zhu et al., 2017). The SD is a modified cell junction that bridges the gap between intercalated foot processes coming from adjacent podocytes and, together with the fenestrated endothelium of glomerular capillaries and the glomerular basement membrane, forms the glomerular filtration barrier. Nowadays, it is assumed that the SD is a crucial component of the barrier and the principal target of agents triggering nephropathies (Grahammer et al., 2013; Kawachi and Fukusumi, 2020; Wiggins, 2007). The main components of this specialised junction are two transmembrane proteins of the immunoglobulin superfamily: nephrin and NEPH1 (which are engaged in homo- and heterotypic interactions across the slit pore through their extracellular domains and contribute to the formation of the molecular filter). Their short cytoplasmic tails associate with multiple partners, including regulators of the cytoskeleton, eliciting signals that are fundamental to regulating podocyte cytoarchitecture, polarity and viability (Barletta et al., 2003; Benzing, 2004; Gerke et al., 2003; Grahammer et al., 2013). Mutations in the main SD components and their associated proteins lead to the collapse of the SD and to massive proteinuria (Boute et al., 2000; Donoviel et al., 2001; Kestilä et al., 1998; Shih et al., 1999; Tryggvason et al., 2006).

Nephrocytes SDs seal the entrance of labyrinthine channels (LChs): membrane ingressions that extend through the whole surface of the nephrocyte and where most of its endocytic activity takes place. At the ultrastructural level, SDs are visualised as double filaments that span the gap at the channel entrance, roughly 40 nm in width. The main components of the nephrocyte SD, Dumbfounded (Duf) and Sticks and stones (Sns), are orthologues of NEPH1 and nephrin, respectively (Weavers et al., 2009; Zhuang et al., 2009). Moreover, these transmembrane proteins associate with intracellular SD components that are also conserved in mammals. For example, Duf binds to Polychaetoid (Pyd), the orthologue of ZO-1, which associates with NEPH1 (Weavers et al., 2009), and SD stability is similarly regulated by phosphorylation (Tutor et al., 2014). Thus, nephrocytes can be considered the fly equivalent of podocytes with regard to ultrafiltration.

In addition to having SDs, nephrocytes also express *Cubilin* (*Cubn*) and *Amnionless* (Zhang et al., 2013). These are orthologues of mammalian cubilin (CUBN) and amnionless (AMN), which are components of the cubilin-amnionless (CUBAM) complex, an endocytic receptor involved in protein reabsorption from the primary filtrate in the renal proximal tubules (Fyfe et al., 2004; Pedersen et al., 2010). RNAi-mediated attenuation studies showed that Cubn together with Amnionless promote protein uptake in nephrocytes (Zhang et al., 2013). This led to the interpretation that nephrocytes could be used to model the vertebrate proximal tubules (Gleixner et al., 2014; Zhang et al., 2013). However, recent studies have shown that *CUBN* and *AMN* are also expressed in murine and human glomerular podocytes (Gianesello et al., 2017; Park et al., 2018; Prabakaran et al., 2012; http://humphreyslab.com/SingleCell/), where they could be involved in receptor-mediated endocytosis of proteins filtered in the glomerulus, such as albumin. This opens the possibility that some of the functions of the CUBAM complex might also be conserved between nephrocytes and podocytes.

CUBN is a highly glycosylated protein consisting of an N-terminal region of 110 amino acids, followed by eight epidermal growth factor (EGF) repeats and 27 CUB (complement-C1r/C1s, Uegf, Bmp1) domains that provides the ligand-binding regions to the complex. AMN is a single-pass transmembrane protein with an ectodomain comprising a SEA (sperm protein, enterokinase and agrin) domain, a cysteine-rich region and two β-helix domains. Its cytoplasmic tail contains two FXNPXF motifs that promote ligand-independent internalisation (Christensen et al., 2012). Previous studies have shown that association of both proteins at the endoplasmic reticulum (ER) is required for trafficking of the CUBAM complex to the plasma membrane (PM), and suggested that CUBN was assembled into trimers (Lindblom et al., 1999), implicating both the N-terminal stretch and the EGF motifs of CUBN in the association with AMN (Coudroy et al., 2005; Fyfe et al., 2004). The recent discovery of the CUBAM structure by X-ray crystallography revealed the nature of this interaction by showing how three CUBN monomers assemble through their N-terminal regions to create a single intermingled β-helix domain that contacts one of the three-faced β-helix domains in AMN; the residues contributing to this association were also determined (Larsen et al., 2018). The EGF motifs are not involved in this association, but they might be fundamental for the proper folding of CUBN that allows trimer formation prior to AMN association.

In this study, we have used *Drosophila* to assess the requirement of the CUBAM endocytic receptor in nephrocyte biology, with an emphasis on SD dynamics. We identify a previously unreported component of the CUBAM receptor, Cubulin2 (*Cubn2*; *CG42255* – FlyBase), that is necessary for correct SD positioning at the entrance of the LChs and for overall nephrocyte architecture. Thus, in the absence of *Cubn*2, nephrocytes lose their characteristic morphological features and supernumerary SDs are found in the interior of the cell that are associated with elongated LChs. Furthermore, by generating loss-of-function alleles for *Drosophila Cubn* and *Amnionless* we reveal that those mutants exhibit similar SD and subcellular architectural phenotypes. Like its paralogue *Cubn*, *Cubn2* is also required for nephrocyte endocytic activity, and we identified *Idgf2* as a putative Cubn2 endogenous ligand. Our observation that in *Drosophila* CUBAM is a tripartite complex formed by Cubn, Cubn2 and Amnionless, led us to explore the possible similarities in the structural organisation of CUBAM complexes in vertebrates and flies. We also show that the activity of the CUBAM receptor is responsible for most clathrin-mediated endocytosis (CME) in nephrocytes and that this endocytic activity is fundamental for SD positioning and nephrocyte global organisation. Moreover, an imbalance between endocytosis and exocytosis appears to be the major driver of CUBAM mutant phenotypes. Exogenous expression in nephrocytes of the endocytic receptor Megalin rescues endocytosis in the absence of CUBAM, but fails to completely restore SD positioning, thus pointing to a specific requirement for the CUBAM receptor in SD recycling.

## RESULTS

### *Cubilin2* is required for nephrocyte morphology, maintenance of SDs at the cell surface and global endocytosis

**Fig. 1.**
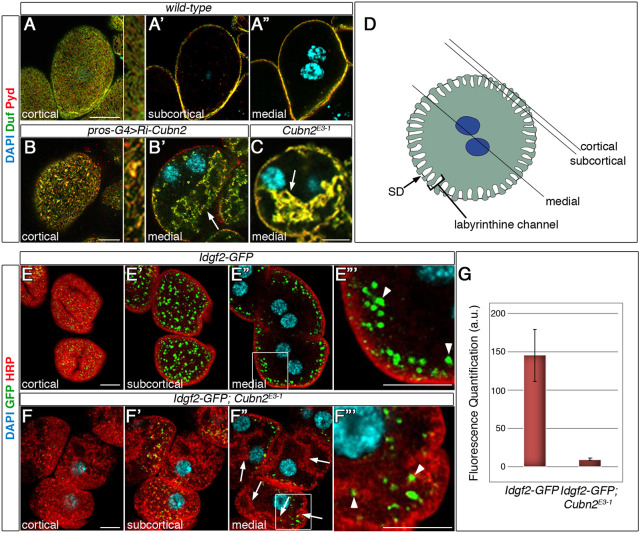
**Cubn2 is required for SD positioning.** Larval nephrocytes. (A-C) Confocal sections. There is fingerprint-like distribution of Duf and Pyd at the external membrane in the wild type (A and detail) (*n*=22 nephrocytes (N)/3 strings (S). In *Cubn2*-RNAi-silenced (B,B′, *n*=43N/4S) and *Cubn2*^E3-1^ nephrocytes (C, *n*=46N/3S) Duf/Pyd density is reduced at the PM (B and detail), both proteins accumulate in internal aggregates (arrows, B′,C) and nuclei are acentric. (D) Nephrocyte scheme indicating cortical, subcortical and medial planes. (E-F‴) Idgf2 uptake (green) in control (E-E‴, *n*=40N/2S) and in *Cubn2^E3-1^* (F-F‴, *n*=28N/3S) *Idgf2-GFP* nephrocytes (boxed areas in E″ are enlarged in E‴ and F‴). In F″,F‴, the scant GFP signal (arrowheads) is associated with HRP-labelled PM ingressions (arrows). In G, GFP fluorescence intensity was quantified in arbitrary units (±s.e.). Details in A,B are at three times higher magnification. Scale bars: 10 µm.

In a genetic screen aimed to uncover genes that affect the stability of the nephrocyte SD using RNAi-mediated attenuation, we identified *CG42255* as being required for correct SD positioning in garland nephrocytes. Thus, by using antibodies against the SD components Dumbfounded (Duf) and Polychaetoid (Pyd), we found a clear reduction in the number of SDs located in the cortical region in its characteristic fingerprint pattern (Fig. 1A,B). Intriguingly, in *CG42255* attenuated cells, Duf and Pyd also colocalised in internal aggregates that occupy large regions of the cytoplasm, which are never found in control nephrocytes (arrow in Fig. 1B′, compare with 1A″, see also Fig. 1D). *CG42255* is a paralogue of *Cubn* and, accordingly, we renamed it *Cubn2*. We used CRISPR/Cas9 to generate novel loss-of-function mutations in *Cubn2*. We obtained four independent alleles, all homozygous viable and selected one for its phenotypic analysis – *Cubn2^E3-1^* – which expresses a Cubn2 protein truncated after the first CUB domain (see Materials and Methods and Fig. S1A,B for details). The distribution of the SD components Duf and Pyd in *Cubn2^E3-1^* is very similar to that observed after *Cubn2* silencing (Fig. 1C).

As *Cubn* and *Amnionless* are involved in protein uptake in nephrocytes (Zhang et al., 2013), we investigated whether *Cubn2* was also required for nephrocyte endocytosis. We first assessed the uptake of dextrans by *Cubn2^E3-1^* nephrocytes, noticing that it was severely compromised (Fig. S2). Then, we measured the uptake of Imaginal disc growth factor 2 (Idgf2), a *Drosophila* trophic factor abundant in the haemolymph that has been suggested to be degraded by nephrocytes (Broz et al., 2017). We found that, in the absence of functional Cubn2, Idgf2 was not internalised because only a faint GFP signal, mostly associated with membrane ingressions, was detected in *Cubn2^E3-1^* nephrocytes (Fig. 1F-F‴,G, compare with Fig. 1E-E‴). These results demonstrate that Cubn2 plays a major role in nephrocyte endocytosis, identifying *Idgf2* as a probable endogenous ligand for this endocytic receptor complex.

**Fig. 2.**
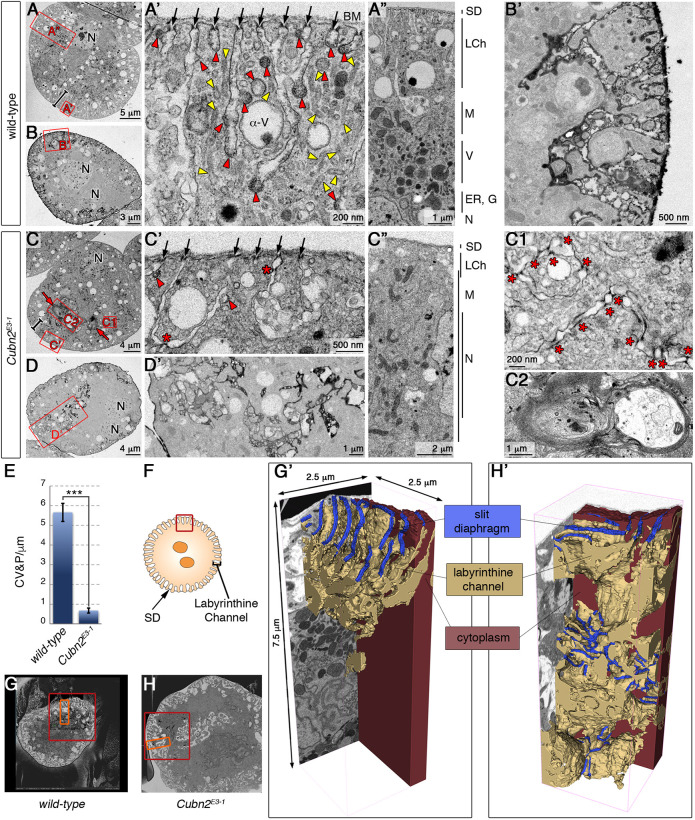
**Ultrastructural analysis of *Cubn2^E3-1^* nephrocytes.** (A-D′) TEM images from medial sections of larval nephrocytes. Bars in A and C indicate the depth of the subcortical zone and red squares indicate the regions enlarged in adjacent panels. (A-A″) Images showing the length of the channels sealed by SDs (arrows), the profusion of clathrin-coated vesicles/pits and tubules (red and yellow arrowheads, respectively), and the characteristic zonation of wild-type cells (A″) (*n*=10). (B,B′) Tannic acid impregnation in wild-type nephrocytes (*n*=3). (C-C2) *Cubn2^E3-1^* nephrocytes display a shortening of the LCh layer (bars, C,C″ with details in C′), scant clathrin-coated structures (arrowheads), reduced SD density at the cortex (black arrows in C′), ectopic SDs in internal regions (stars in C′,C1) and the presence of multilaminar structures (C, red arrows and C2) (*n*=11). (D,D′) A tannic acid-impregnated *Cubn2^E3-1^* nephrocyte revealing the continuity of the membrane ingressions with the extracellular space (*n*=8). (E) Quantification of clathrin-coated structures in wild-type and *Cubn2^E3-1^* nephrocytes (*n*=60 and 100 micrographs, data are mean±s.e.m.; *P*<0.05). (F) Scheme of the region used for FIB-SEM analysis. (G,H) Low-magnification images of wild-type (G) and *Cubn2^E3-1^* (H) nephrocytes selected for FIB-SEM acquisition; red squares show regions processed by FIB-SEM and orange rectangles show those used for segmentation. (G′,H′) 3D models of LCh and SD distribution in wild-type (G′) and *Cubn2^E3-1^* (H′) nephrocytes. The LCh model was partially sectioned to allow visualisation of internal regions. SDs cover 35% of the nephrocyte surface in the wild type and 16% in *Cubn2^E3-1^*. BM, basement membrane; M, mitochondria; V, vacuole; G, golgi; N, nucleus; LCh, labyrinthine channel; SD, slit diaphragm.

To examine in more detail the Duf-Pyd internal aggregates of *Cubn2^E3-1^* nephrocytes, we performed ultrastructural analysis by transmission electron microscopy (TEM). In contrast to the characteristic arrangement of cellular features in defined concentric layers observed in the wild-type (Fig. 2A-A″), those layers were poorly defined in *Cubn2^E3-1^* nephrocytes (Fig. 2C-C″). Thus, the cortical region contains fewer SDs (arrows in Fig. 2A′,C′) and SDs are not strictly confined to the entrance of the LChs but are also found at internal ectopic positions (asterisks in Fig. 2C′). The subcortical region occupied by LChs, clathrin-coated vesicles and pits, tubules, and α-vacuoles is less compact in the mutants (bars in Fig. 2A,C) Remarkably, clathrin-coated structures and tubules, which are very abundant in the wild type, are rare in *Cubn2^E3-1^* nephrocytes (Fig. 2A′,C′, quantified in Fig. 2E). This indicates that CME is severely compromised in *Cubn2* mutants. Additionally, the subcortical region seems to be continuous with large areas of the cytoplasm occupied by membrane ingressions containing SD-like densities (asterisks in Fig. 2C1), which correlate with the extensive accumulation of Duf and Pyd in the interior of the nephrocytes (Fig. 1B′,C). Presence of such ectopic SD-like densities and tannic acid impregnation in advance of TEM, which showed that the ingressions were continuous to the extracellular space (compare Fig. 2B-B′ with D-D′), suggested them to be elongated LChs. Furthermore, in mutant nephrocytes, vacuoles and mitochondria are regularly distributed throughout the cytoplasm instead of being confined to the transition zone (Fig. 2A,C, details in A″,C″), the nuclei are displaced from their central position (N in Fig. 2, Fig. S2C) and multilaminar structures are frequently observed (arrows in Fig. 2C, detail in C2), which have previously been described as aberrant lysosomes (Chang et al., 2002; Dermaut et al., 2005; Psathaki et al., 2018).

To better understand the organisation of the membrane ingressions and associated SD-like densities, we resorted to focussed ion beam/scanning electron microscopy (FIB-SEM) to obtain a three-dimensional (3D) ultrastructural representation of the cortical and subcortical regions in *Cubn2^E3-1^* and wild-type nephrocytes (Fig. 2F-H′ and Movies 1 and 2). The 3D models clearly showed the absence of tubular elements at the subcortical region, the scarcity of SDs at the cell surface and the presence of abundant ingression of membranes towards deeper regions, which maintain continuity with the PM in mutant nephrocytes. Numerous rows of SD-like densities very similar to the rows of SDs found at the nephrocyte cortical region, associate with these internalised membranes and seal them at multiple locations, forming a network of interconnected vesicles with many shapes. The ultrastructural similarity between the internalised SD-like densities and the normal SDs, together with the detection of internalised SD proteins Duf and Pyd in *Cubn2* mutants (Fig. 1B,C), strongly suggests that these SD-like densities are indeed mislocalised SDs and that Cubn2 is essential to control SDs localisation in nephrocytes.

### Cubilin, Cubilin-2 and Amnionless form a tripartite CUBAM receptor in *Drosophila*

**Fig. 3.**
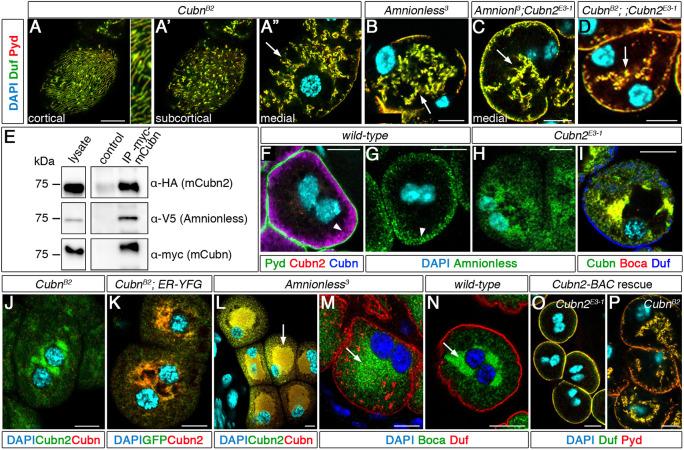
**CUBAM is a tripartite complex in nephrocytes.** Larval nephrocytes. (A-D) Duf/Pyd distribution in *Cubn^B2^* [A-A″, detail is at 3× higher magnification; *n*=47 nephrocytes (N)/3 strings (S)], *Amnionless^3^* (B, *n*=36N/3S), *Amnionless^3^;Cubn2^E3-1^* (C, *n*=5N/1S) and *Cubn^B2^;;Cubn2^E3-1^* (D, *n*=38N/2S) nephrocytes is undistinguishable from that of *Cubn2^E3-1^*; arrows indicate accumulation of Duf/Pyd in internal aggregates; note the acentric nuclei. (E) Immunoprecipitations from extracts ectopically expressing mini-Cubn-Myc, mini-Cubn2-HA and Amnionless-V5. Both mini-Cubn2 and Amnionless co-immunoprecipitate with mini-Cubn (lane 3). Compare with control (lane 2) immunoprecipitated with unspecific IgGs. (F,G) Cubn and Cubn2 colocalise at the LCh membrane (F, arrowhead, *n*=21N/2S), where Amnionless is localised (G, arrowhead, *n*=40N/2S). (H,I) In *Cubn2^E3-1^* nephrocytes, Amnionless is retained in the ER, which is labelled with Boca (H, *n*=42N/3S; I, *n*=10N/6S). (J,K) Cubn2 ER retention in *Cubn^B2^* nephrocytes (J, *n*=54N/5S; K, *n*=41N/4S). (L-N) In *Amnionless^3^*, both Cubn and Cubn2 are retained in an oversized ER (arrows in L, M, compare with N) (L, *n*=15N/2S; M, *n*=32N/2S; N, *n*=30/2S). (O,P) *Cubn2^E3-1^* (*n*=52N/4S) but not *Cubn^B2^* (*n*= 43N/4S) nephrocyte mutant phenotypes are rescued by genomic BAC CH322-175O13. Scale bars: 10µm.

None of the structural features of *Cubn2* mutant nephrocytes mentioned above have been described for *Cubn* or *Amnionless* (Zhang et al., 2013), raising the issue of whether there are divergent functions specific to *Cubn2*. Therefore, we decided to re-examine the phenotypes of *Amnionless* and *Cubn* mutants. We induced novel loss-of-function alleles and selected *Cubn^B2^* for analyses, a truncation removing most of the C-terminal region of Cubn (including the EGF repeats), and *Amnionless^3^*, which lacks the intracellular, transmembrane and most of the extracellular domains of Amnionless (Fig. S1C,D). Both alleles are homozygous viable. Notably, we found that their phenotypes were very similar to that of *Cubn2* mutants with respect to SD localisation, with reduced density at cortical regions (Fig. 3A, compare with Fig. 1A,B) and strong accumulation in internal membranes (Fig. 3A′-B). Both alleles also show acentric nuclei (Fig. S2C), indicating an effect on global subcellular architecture. These data are consistent with the interpretation that Cubn2 participates in the same endocytic complex as Cubn and Amnionless. As a first approach to examine this possibility, we performed co-immunoprecipitation assays. We generated mini-Cubn-Myc and mini-Cubn2-HA proteins containing intact N-terminal domains and the eight EGF repeats. A similar region in mammalian cubilin was described to be sufficient for its assembly with AMN and for trafficking to the PM (Coudroy et al., 2005). The mini-Cubilin proteins were expressed together with V5-tagged Amnionless and precipitated with an anti-Myc antibody. These assays revealed that mini-Cubn2 and Amnionless co-immunoprecipitated with mini-Cubn, suggesting that they are part of the same complex (Fig. 3E).

**Fig. 4.**
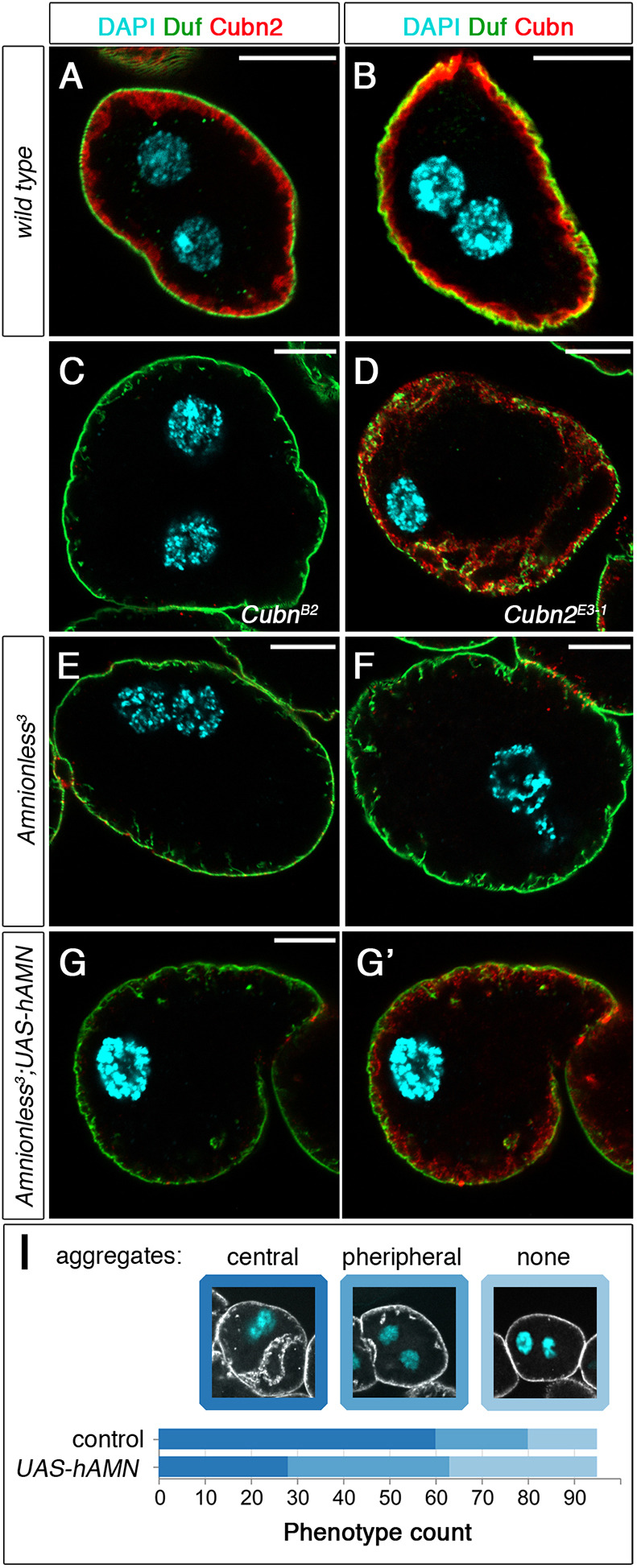
**Extracellular labelling of CUBAM proteins in larval nephrocytes.** (A-G′) SDs revealed with anti-Duf. (A,B) In wild-type nephrocytes, both Cubn2 (A, *n*=12 out of 12 nephrocytes) and Cubn (B, *n*=105 out of 113N) accumulate at the LCh membrane. (C,D) Although Cubn2 does not reach the LCh membrane in *Cubn^B2^* (C, *n*=15 out of 15N), some of Cubn does in *Cubn2^E3-1^* nephrocytes (D, *n*=13 out of 13N). (E,F) Neither Cubn2 (E, *n*=7 out of 7N) nor Cubn (F, *n*=6 out of 6N) reaches the LCh membrane in *Amnionless^3^* mutants. (G,G′) In *Amnionless^3^* nephrocytes expressing h-AMN (*pros-*GAL4), a small fraction of Cubn (G′), but not Cubn2 (G), reaches the LCh membrane (*n*=10 out of 15N). All mutant genotypes have acentric nuclei. (I) hAMN-Myc expression slightly ameliorates the *Amnionless^3^* phenotype. Quantification of the rescue effect of hAMN-Myc (*pros-GAL4*) on SD positioning in *Amnionless^3^* nephrocytes, evaluated by the presence of SD proteins in aggregates located centrally (stronger phenotype) or peripherally (weaker phenotype). Classification was carried out using full cell image stacks. Representative confocal sections corresponding to each class are shown for reference, *n*=95N/19 strings for each genotype. Scale bars: 10 µm.

To further verify the formation of a Cubn/Cubn2/Amnionless complex, we used specific antibodies to analyse the distribution of the three proteins, finding that they colocalise at the subcortical region of larval garland nephrocytes occupied by the LChs, a region of massive endocytosis (Fig. 3F,G). In mammals CUBN and AMN require each other to traffic to the membrane (Coudroy et al., 2005; Udagawa et al., 2018). Therefore if Cubn2 is a subunit of the CUBAM receptor, we anticipated that it might similarly require both Amnionless and Cubn for its trafficking. In agreement with this hypothesis, we found that, in *Cubn^B2^* mutants, Cubn2 protein is minimally present at the subcortical region and instead accumulates in large discrete aggregates that, by their position and colocalisation with the marker sqh-ER-YFP, correspond to the ER (Fig. 3J,K). Moreover, Cubn2 was not detected at the extracellular surface of non-permeabilised *Cubn^B2^* nephrocytes, in contrast to the strong signal obtained for the wild type (Fig. 4A,C), demonstrating lack of trafficking to the PM. Similarly, in *Cubn2^E3-1^* mutants, Amnionless and Cubn proteins accumulate in the ER and are mostly absent from the LCh-rich subcortical region (Fig. 3H,I). However, low levels of Cubn were detected at the cell surface of non-permeabilised *Cubn2^E3-1^* cells, indicating that a minor fraction of Cubn can reach the PM in this genetic background (Fig. 4B,D). This is in agreement with findings in mammals showing that a C-terminal truncated CUBN protein lacking all CUB domains but still containing EGF1-8 domains, which are also present in the *Cubn2^E3-1^* mutant protein, can mediate partial trafficking of the CUBAM complex to the PM (Coudroy et al., 2005). Finally, in *Amnionless^3^* mutants, both Cubn and Cubn2 accumulate in an oversized ER and are absent from the subcortical region (Fig. 3L-N). In addition, these proteins were not detected at the extracellular surface of non-permeabilised cells (Fig. 4E,F).

The previous results demonstrate that Cubn, Cubn2 and Amnionless require each other to traffic to the PM and support the hypothesis that they are subunits of a single CUBAM complex. If this were the case, we would expect the phenotypes caused by mutations in any of the three components or by the double mutant combinations to be identical. Accordingly, the phenotypes of mutations affecting members of the CUBAM receptor (Figs 1B,C and 3A,B) were not aggravated in the double mutants *Amnionless^3^; Cubn2^E3-1^* or *Cubn^B2^;; Cubn2^E3-1^* (Fig. 3C,D). Interestingly, although reintroduction of the *Cubn2* genomic region (BAC clone CH322-175O13) completely rescued the nephrocyte phenotype of *Cubn2^E3-1^* individuals, it failed to rescue *Cubn^B2^* mutants (Fig. 3O,P). Taken together, these results demonstrate that Cubn and Cubn2 are not functionally redundant and that, in *Drosophila*, the CUBAM complex is a tripartite receptor composed of Amnionless and two Cubilin proteins, all of which are essential for promoting endocytosis and for the correct positioning of SD components in nephrocytes.

### Structural organisation of the CUBAM receptor in *Drosophila melanogaster*

The mammalian CUBAM complex contains a single AMN subunit associated with a CUBN homotrimer (Larsen et al., 2018). Our finding that, in *Drosophila*, the complex contains two different Cubilin subunits indicates a substantial divergence from its mammalian counterpart and poses interesting questions about its structural organisation. In this regard, it was shown that human amnionless (hAMN) could partially restore protein uptake in *Amnionless*-attenuated nephrocytes (Zhang et al., 2013), suggesting that hAMN can still interact with *Drosophila* Cubn and/or Cubn2 and generate functional complexes. Thus, we decided to examine this possibility further by analysing whether hAMN can promote the correct positioning of SDs and the trafficking of Cubn or Cubn2 in *Amnionless^3^* mutants. We compared *Amnionless^3^* larvae expressing hAMN in nephrocytes with their siblings from the same cross that did not express the transgene. Our results revealed that although hAMN is unable to replace Amnionless fully, there was some correction of the SD internalisation phenotype (Fig. 4I). Then, we examined whether Cubn or Cubn2 proteins could reach the PM in non-permeabilised *Amnionless^3^* nephrocytes expressing hAMN. We detected Cubn but not Cubn2 at the PM, suggesting that hAMN is able to form a CUBAM complex only with *Drosophila* Cubn (Fig. 4G,G′). In agreement with this interpretation, we found that when hAMN was expressed in wild-type, *Cubn^B2^* or *Cubn^B2^*; *Amnionless^3^* double mutant nephrocytes, hAMN was retained in the ER (Fig. S3A,E,F). In contrast, in *Amnionless^3^*, *Cubn2^E3-1^* or *Amnionless^3^*; *Cubn2^E3-1^* double mutants, hAMN was detected a low levels in the subcortical region and in association with internalised SDs, suggesting it reached the PM, possibly in a complex with Cubn (Fig. S3B-D). To account for these results, we propose that hAMN, in the absence of endogenous Amnionless, can interact at low affinity with Cubn, but not with Cubn2 in the ER, assembling a functional hybrid complex that would traffic to the PM, even though in this background most of Cubn remains retained in the ER. This is consistent with the fact that the region in human CUBN involved in the interaction with AMN is partially conserved in *Drosophila* Cubn, but not in Cubn2 (Fig. S3G, red bar).

Interestingly, a phylogenetic analysis suggests that this particular organisation of the CUBAM complex might not be unique to *Drosophila*. Indeed, although most species for which cubilin orthologues can be identified contain a single copy, two or even three cubilin paralogues are present in most fly species and in some aphids, hymenopterans, beetles and bony fishes (Fig. S4).

### An endocytosis blockade in CUBAM mutant nephrocytes is responsible for their morphological disorganisation and SD mispositioning phenotype

**Fig. 5.**
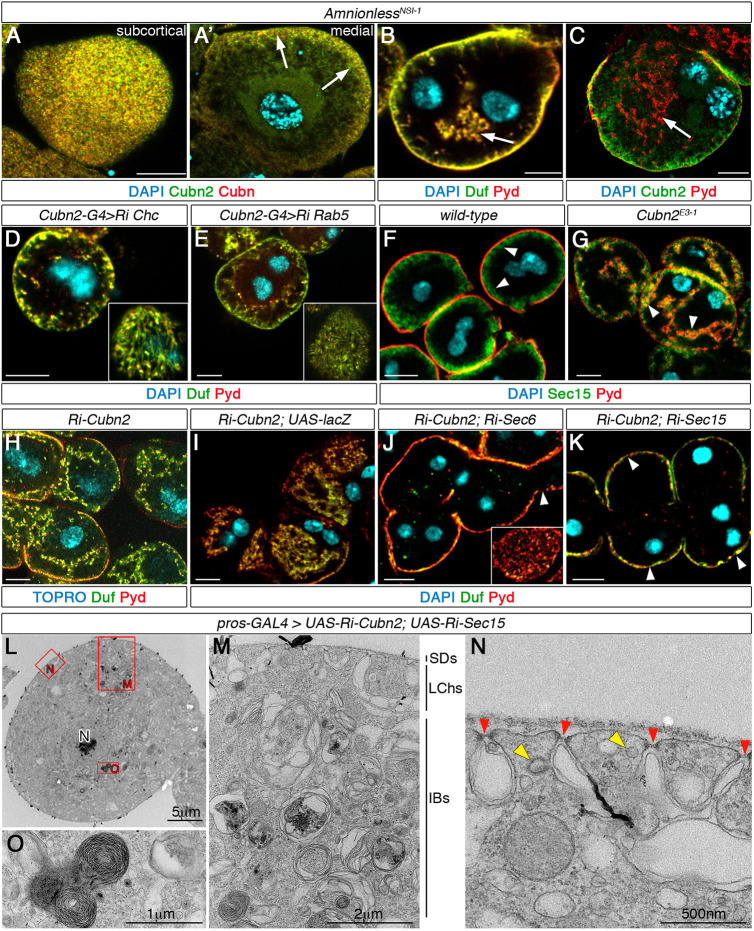
**Imbalance of endocytic and exocytic activities leads to SD mispositioning.** Larval nephrocytes. (A-C) Deletion of the FXNPXF-internalisation domain in *Amnionless^NSI-1^* sustains Cubn and Cubn2 trafficking to the membrane [A, A′, arrows, *n*=19 nephrocytes (N)/3 strings(S)] and induces internal aggregates of Duf/Pyd (arrows in B, *n*=84N/6S, and C *n*=25N/3S) and nuclei mispositioning. (D,E) Attenuation of endocytosis by Chc (D, *n*=55N/7S) or Rab5 (E, *n*=87N/10S) silencing leads to internal aggregates of SD components and decreased SD density at the cortical region (insets). (F,G) Sec15 accumulates at the LCh membrane in wild type (F, arrowheads, *n*=19N/2S) and additionally in internal membranes in *Cubn2^E3-1^* nephrocytes (G, arrowheads, *n*=17N/2S). (H-K) Attenuation of exocytosis by depleting either Sec6 (J, *n*=30N/3S) or Sec15 (K, *n*=86N/6S) (*pros-GAL4*) corrects the ingression of SD-bearing membranes induced by *Cubn2* silencing (H, *n*=38N/3S; titration control in I, *n*=23N/2S) but not the lower SD density at the external membrane (J,K, inset and arrowheads). (L-O) Medial TEM sections of nephrocytes simultaneously attenuated for *Cubn2* and *sec15*. Internal SDs are absent, SD density is reduced (N, red arrowheads), tubules and clathrin-coated structures are scarce (N, yellow arrowheads) and the multilaminar structures are present (O, *n*=8N). Scale bars: 10 µm in A-K.

This and previous reports (Zhang et al., 2013; Hermle et al., 2016) indicate that CUBAM members are involved in protein uptake in nephrocytes. Furthermore, we have shown a dramatic reduction in clathrin-coated structures in *Cubn2^E3-1^* nephrocytes, indicating a strong inhibition of CME (Fig. 2E). Considering these observations, we investigated whether the morphological disorganisation and SD mispositioning phenotypes observed in CUBAM-defective nephrocytes could be solely caused by a blockade in endocytosis or whether the failure of CUBAM proteins to traffic to the membrane and their abnormal retention in the ER could also contribute. To this end, we generated a novel *Amnionless* mutant, *Amnionless^NSI-1^*, which produces an Amnionless truncated protein lacking the FXNPXF domain required for ligand-independent internalisation, but retaining the three-faced β-helix and the transmembrane domains required in vertebrates for docking of cubilin trimers and their traffic to the membrane (Fig. S1A,E). As expected, we observed that in *Amnionless^NSI-1^* nephrocytes most of Cubn and Cubn2 exited the ER and trafficked to the LCh membrane (Fig. 5A,C, arrows in A′). However, *Amnionless^NSI-1^* mutants still exhibited aberrant distribution of SDs in internal membranes and acentric nuclei (Figs. 5B,C, Fig. S2C), indicating that these phenotypes are directly associated with a deficit in endocytosis. In agreement with this interpretation, we found that reducing endocytosis by attenuation of *Clathrin heavy chain* (*Chc*) or *Rab5* (Fig. 5D,E), or in *dor^8^* mutants lacking the *Drosophila* CORVET and HOPS function (Lőrincz et al., 2016) produced similar phenotypes (Fig. S5A). However, attenuation of *Rab1* or mutations in *light*, a *Drosophila* component of the late endosomal tether HOPS, did not induce the ingression of SDs (Fig. S5B,C).

### Restoration of the homeostatic membrane balance in CUBAM mutant nephrocytes ameliorates the SD delocalisation phenotype

In neurons and neuroendocrine cells, which display high endocytic and exocytic activities like the nephrocytes, these two processes are tightly regulated to ensure membrane balance homeostasis (Houy et al., 2013; Liang et al., 2017). Thus, a plausible interpretation of CUBAM phenotypes is that the excess of PM and SD ingressions observed are a consequence of the imbalance between endocytosis and exocytosis. To test this hypothesis, we took two complementary approaches aimed at restoring the membrane balance in *Cubn2* mutant nephrocytes. First, we inhibited exocytosis by genetic attenuation of the exocyst components Sec6 and Sec15 (Mei et al., 2018; Wu and Guo, 2015). Using an anti-Sec15 antibody, we found that this exocyst protein accumulates as expected at the subcortical region of wild-type and *Cubn2* mutant nephrocytes, and also at the membrane ingressions in *Cubn2* mutants (arrowheads in Fig. 5F,G). Next, we simultaneously depleted CUBAM-mediated endocytosis and general exocytosis by expressing the transgenic lines *UAS-RNAi-sec6* (or *UAS-RNAi-sec15*) and *UAS-RNAi-Cubn2* under the control of *pros-GAL4*. As an internal control, we co-expressed *UAS-RNAi-Cubn2* and *UAS-lacZ* (Fig. 5I, compare with H) to exclude possible phenotype suppression due to GAL4 titration. We found that the simultaneous attenuation of *Cubn2* and *sec6* or *sec15* (Fig. 5J,K) completely suppressed the ingression of SDs without recovering the SD shortage at the cortical region (inset in Fig. 5J and arrowheads in J,K) and only partially rescued the nuclear delocalisation phenotype (Fig. S2C). These observations were further confirmed by TEM analyses of nephrocytes simultaneously attenuated for *Cubn2* and *sec15* (Fig. 5L-O), which do not display SD ingressions (Fig. 5M). However, note the lower density of SDs (red arrowheads in Fig. 5N), the scarcity of clathrin-coated vesicles and pits, tubules and clear vacuoles (Fig. 5N) and the presence of large multilaminar organelles (Fig. 5M,O).

**Fig. 6.**
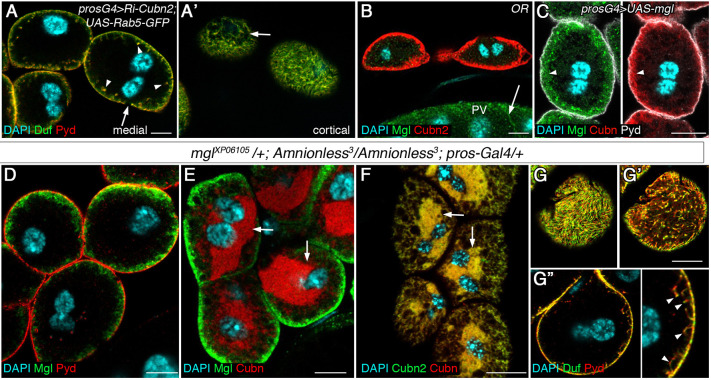
**Restoration of endocytosis corrects SD delocalisation in *Cubn2^E3-1^* nephrocytes.** (A,A′) Promoting global endocytosis by Rab5 expression in *Cubn2-*attenuated nephrocytes largely restores SD positioning [arrowheads indicate SD internal accumulation; arrows indicate gaps in SD-protein distribution; *n*=106 out of 131 nephrocytes (N), 22 strings (S) examined. (B) Megalin expression in wild-type nephrocytes [arrow indicates Mgl in the proventriculus (PV), *n*=29N/3S]. (C) Ectopically expressed Mgl accumulates with Cubn in the LCh membrane (arrowheads, *n*=20N/2S). (D-G″) Ectopic Mgl in *Amnionless^3^* nephrocytes localises at the LCh (D, *n*=21N/2S,) concomitant with the ER retention of Cubn and Cubn2 (arrows in E, *n*=42N/4S, and F, *n*=18N/2S), and suppresses the Duf/Pyd internal aggregates found in *Amnionless^3^* (G″, *n*=28/2S). However, note the lower density of SDs at the periphery (G, compare with Fig. 1A) and the presence of ectopic SDs at the subcortical region (G′, arrowheads in G″; inset in G″ is at 2.5× higher magnification). Scale bars: 10µm.

In a second approach to regain membrane balance, we promoted endocytosis in *Cubn2* mutant nephrocytes, either by overexpression of Rab5 or by ectopic expression of an unrelated endocytic receptor. Overexpression of Rab5 was described to promote both receptor-mediated endocytosis and fluid-phase endocytosis (Bucci et al., 1992; Grbovic et al., 2003). We found that the simultaneous expression of *UAS-RNAi-Cubn2* and *UAS-Rab5-GFP* in nephrocytes largely corrected the deep ingressions of SDs (Fig. 6A,A′). To promote receptor-mediated endocytosis, we chose Megalin (Mgl), the *Drosophila* orthologue of mammalian megalin/LRP2 that is involved in tubular reabsorption of multiple ligands and that has been shown to promote endocytosis in *Drosophila* (Gleixner et al., 2014; Riedel et al., 2011). Mgl is undetectable in nephrocytes (Fig. 6B), but it accumulates at high levels with Cubn in LChs when ectopically expressed in these cells, although Mgl is also present in vesicles (arrowheads in Fig. 6C). We first validated that Mgl ectopic expression in *Amnionless^3^* nephrocytes induces an increase in dextran uptake (Fig. S6). Importantly, Mgl expression rescued the deep ingressions of membranes and SDs (Fig. 6D, compare with Fig. 3B). However, it did not rescue other phenotypic features of *Amnionless* mutants, such as the presence of an oversised ER detected by Cubn and Cubn2 accumulation (Fig. 6E,F), the acentric nuclear position (Fig. S2C) and, notably, the ectopic location of SDs at the subcortical region, from where they are excluded in the wild type (Fig. 6G-G″, compare with Fig. 1A-A″).

Taking into account all the results mentioned above, the restoration of the membrane balance in CUBAM mutant nephrocytes, either by inhibiting exocytosis or by promoting endocytosis, rescues the abnormal ingressions of the PM. However, other aspects of the nephrocyte cellular organisation, such as nuclei positioning, are not corrected.

Furthermore, although increasing endocytosis by either Rab5 or Mgl overexpression restores receptor-mediated endocytosis and largely eliminates the accumulation of ectopic SDs in the interior of nephrocytes, ectopic SDs can still be detected at the subcortical region, which together with the absence of tubular elements in this region (see Discussion) could uncover a hitherto unsuspected function of CUBAM in regulating SD dynamics.

## DISCUSSION

A major finding of this study is the identification of Cubn, its paralogue Cubn2 and Amnionless as necessary for the correct positioning of SDs. As was previously shown for Cubn and Amnionless (Zhang et al., 2013), we demonstrate that Cubn2 functions as an endocytic receptor and that Cubn2, Cubn and Amnionless form a tripartite endocytic complex in *Drosophila*. We provide additional evidence for a major impact of receptor-mediated endocytosis on nephrocyte cytoarchitecture, and emphasise the relevance of maintaining the balance between endocytic and exocytic activities in these excretory cells for preserving their subcellular organisation, including SD positioning. The observation that the endocytic receptor Megalin/LRP2 fails to substitute CUBAM in fully restoring SD positioning despite recovering CME, leads to the most unexpected finding of the present study: the possible direct involvement of the CUBAM receptor in SD recycling. These findings are in line with the reported expression of CUBN and AMN in glomerular podocytes (Gianesello et al., 2017; Park et al., 2018; Prabakaran et al., 2012; http://humphreyslab.com/SingleCell/), and argue for a possible conserved role of the endocytic receptor CUBAM in regulating SD remodelling.

### Phylogenetic analysis of cubilin proteins

The finding that *Drosophila* CUBAM comprises two Cubn paralogues prompted us to explore for CUBN paralogues in other organisms. Most species, including the majority of vertebrates and insects have a single *cubilin* gene, a notable exception being fly species, which have two paralogues. Thus, it appears that a gene duplication took place at the base of the fly lineage giving rise to *Cubn* and *Cubn2*. Independent duplications of cubilin genes also occurred in other species, including aphids, hymenopterans, beetles and bony fishes. Besides, additional duplications resulting in having two *Cubn* and one *Cubn2* genes or one *Cubn* and two *Cubn2* genes also occurred in some fly species (Fig. S4).

The fact that cubilin gene duplications occurred multiple times during evolution suggests the existence of evolutionary pressure to expand the set of potential CUBAM ligands in these organisms. We hypothesise that soon after the duplication and divergence of the parental cubilin gene, cubilin homotrimers and heterotrimers would have co-existed in the cell, with the heterotrimers covering a broader set of potential ligands. However, both homomeric and heteromeric cubilin trimers need to maintain their interaction with AMN in order to reach the PM and be functional, constraining the possibilities for evolutionary changes. The recent identification of the residues involved in the interaction between AMN and CUBN homotrimers indicated that residues in equivalent positions in each CUBN monomer contact different residues in AMN (Larsen et al., 2018). Thus, it is likely that mutations in residues at the CUBN-AMN interface that are either neutral or improve the association between AMN and cubilin heterotrimers would most probably be detrimental for the interaction with the homotrimers, leading to reduced PM exposition and the eventual loss of the homotrimers. This would lead to what we find in *Drosophila*, where only Cubn/Cubn2 heterotrimers are functional. Indeed, the results we obtained after overexpression of hAMN in nephrocytes support this hypothesis. Thus, hAMN, in the absence of endogenous Amnionless, mediates the trafficking of a small fraction of Cubn, but not of Cubn2, from the ER to the PM (Fig. S3), suggesting that hAMN, under these artificial conditions, can only interact with Cubn homotrimers and with very low efficiency. This is consistent with the divergence observed in Cubn2 residues at the interface region (Fig. S3G). It also implies a concomitant evolution of Amnionless to accommodate to these changes, explaining the failure of hAMN to fully substitute for Amnionless (Fig. 4I).

### The impact of CUBAM on SD positioning

Despite the differences in the composition of the CUBAM complex between *Drosophila* and vertebrates, its function in protein uptake is evolutionarily conserved (Zhang et al., 2013). Furthermore, our data indicate that, as in vertebrates, in *Drosophila*, CUBAM is formed by the association of a single Amnionless molecule with a trimer of cubilin monomers that comprise both Cubn and Cubn2 with unknown stoichiometry. Several facts support this interpretation: (1) both Cubn2 and Amnionless co-immunoprecipitate with Cubn; (2) the three components colocalise at the LCh membrane and have similar LOF phenotypes; (3) the three components are mutually dependent for their trafficking to the PM; (4) the N-terminal domains of Cubn/Cubn2 contribute to their association with Amnionless; and (5) hAMN has the ability to form, albeit with very low affinity, a chimeric CUBAM complex with Cubn, suggesting that Cubn can associate as a homotrimer with hAMN. Therefore, a significant part of the conclusions emerging from our studies regarding the impact of CUBAM on SD dynamics might also apply to vertebrates, especially considering the reported expression of CUBAM in podocytes.

A key finding of our study is that CUBAM mediates a significant fraction of global receptor-mediated endocytosis in nephrocytes, which in turn accounts for most endocytosis taking place in these cells, in agreement with previously published data for Cubn and Amnionless (Zhang et al., 2013). Thus, in mutants for any of the CUBAM components, the number of clathrin-coated structures and tubular elements, both very abundant in the wild type, are strongly reduced. Tubular elements in insect nephrocytes are somehow connected to α-vacuoles (Crossley, 1972; Koenig and Ikeda, 1990). Interestingly, tubules with the same morphological characteristics are very abundant in the apical region of renal proximal tubules, where they have been proposed to represent an intracellular early endosomal compartment involved in the recycling of internalised receptor-ligand complexes from endocytic vacuoles back to the external membrane (Hatae et al., 1997). Based on these observations, it might be argued that nephrocyte tubules are similarly involved in quick recycling of receptor-ligand complexes. This interpretation is compatible with experimental evidence showing the extremely fast vanishing rate of tubular elements and α-vacuoles in nephrocytes when endocytosis is temporary blocked using the temperature-sensitive dynamin mutant *shibire^ts^* (Koenig and Ikeda, 1990). It also explains the lack of tubules in *Cubn2* mutants.

We also find that the elimination of CUBAM-mediated endocytosis has a strong impact in the global organisation of nephrocytes, affecting their characteristic organelle zonation and displacing the nuclei from their medial location. We provide previously undocumented evidence of the presence of deep membrane ingressions, which remain in contact with the extracellular space and contain electron-dense material consisting of SD rows similar to those present at the nephrocyte surface. Other aberrant morphological features in CUBAM mutants are the enlargement of the ER compartment, most exacerbated in *Amnionless^3^* mutants, and the accumulation of multilamellar inclusions, which have been described as secondary enlarged lysosomes in the process of clearing autophagic cargo as a result of endocytic defects (Dermaut et al., 2005).

**Fig. 7.**
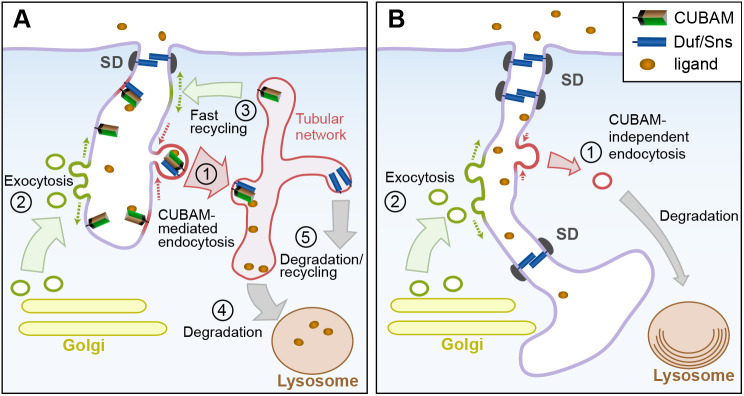
**Model depicting the hypothesised functions of CUBAM in nephrocytes and their impact on SD dynamics.** (A) In wild-type nephrocytes, the CUBAM receptor localised at the LCh membrane mediates the endocytosis of multiple ligands via clathrin-coated vesicles (1). These vesicles fuse into the sorting endosome compartment, which in nephrocytes displays a tubular network aspect. CUBAM molecules are recycled back to the LChs in vesicles, helping restore membrane balance (3), and ligands are routed into the lysosome pathway for degradation (4) or are recycled (5). PM deficit caused by robust CUBAM-mediated endocytosis (1) is compensated for by membrane contributions from exocytosis (2) and from the fast recycling route (3). We hypothesise that CUBAM also facilitates the removal of SD components that were targeted for degradation or recycling (1). (B) When CUBAM-mediated endocytosis is impaired, e.g. as in CUBAM mutants, CME is strongly reduced and only residual CUBAM-independent endocytosis occurs (1). The resulting imbalance between membrane contributions by exocytosis (2) and membrane removal by endocytosis (1) promotes the increase of LCh membranes. This effect, together with a reduction in CUBAM-mediated endocytosis of damaged SD components, can lead to the observed accumulation of additional SDs at subapical and deeper regions of the enlarged LChs. Multilamellar onion-like lysosomes are also represented. Cellular compartments are not to scale.

Given the role of CUBAM in receptor-mediated endocytosis (Zhang et al., 2013, this study), it is plausible that the observed phenotypes could be directly associated with nephrocyte endocytic defects. Indeed, the results obtained for *Amnionless^NSI-1^*, which lacks CUBAM-mediated endocytosis without affecting the localisation of the complex, are in complete agreement with this interpretation. Therefore, the issue is how the blockade of receptor-mediated endocytosis has such a drastic effect on nephrocyte architecture. Nephrocytes display high endocytic and exocytic activities that profoundly shape their morphology. Several lines of evidence point to an imbalance in these activities as the main driver of the phenotypes observed in CUBAM-deficient nephrocytes. First, inactivating Dynamin using the temperature-sensitive allele *shibire^ts1^*, blocked endocytosis and induced the elongation of the LChs (Kosaka and Ikeda, 1983). In addition, we show that silencing *Chc* or reducing *Rab5* activity, but not blocking late endosome maturation into lysosomes in *light* mutants, induced the ingression of membranes bearing SDs, reminiscent of the reported CUBAM phenotypes. Thus, blocking global endocytosis is the main factor responsible for the huge membrane ingressions and SD delocalisation in nephrocytes. A recent report by Wang et al. (2021) described similar internal accumulation of SD components after attenuation of genes involved in CME, and although they interpreted these observations as if the SD components were contained in cytoplasmic granules or vesicles, this is not supported by our ultrastructural analysis. Second, the simultaneous attenuation of *Cubn2* and the exocyst components *sec6* or *sec15* suppressed the SD-bearing PM ingressions without recovering the reduced density of SD at the cortical zone. Furthermore, under these conditions ultrastructural analyses revealed morphological traits indicative of defective endocytosis, such as the strong reduction of clathrin-coated vesicles and pits, tubules and clear vacuoles, coinciding with the presence of autolysosomes/autophagosomes. Finally, promoting global or receptor-mediated endocytosis in the absence of a functional CUBAM receptor has a similar recovery of membrane and SD ingressions. These observations are compatible with the notion that the membrane ingressions are the result of continuous exocytosis in the absence of compensatory endocytosis and emphasise the importance of maintaining the balance between endocytosis and exocytosis to preserve nephrocyte morphology (Fig. 7).

Interestingly, we noticed that, even though Mgl or Rab5 overexpression rescues the extensive membrane ingressions and internal SD aggregates of CUBAM mutants, other aspects of the phenotype were not corrected. In particular, SD rows at the cortical region are more scattered than in the wild type and abundant ectopic SDs sealed the LChs multiple times along a few micrometres-long segments near the cell surface, compared with the single SD found in the wild type. These observations favour the interpretation that the CUBAM receptor probably plays a direct role in SD dynamics, possibly by promoting the endocytosis of SDs that had been targeted for degradation by other processes such as phosphorylation (Fig. 7). In vertebrates, it has been proposed that CME of nephrin after phosphorylation may regulate its steady-state recycling, indicating a relevant role for receptor-mediated endocytosis in SD remodelling (Espiritu et al., 2019; Martin et al., 2020). A relevant question is whether or not CUBAM receptor might participate in vertebrate SD recycling. Many studies have addressed the clinical relevance of mutations in human CUBAM, and recently it has been shown that individuals with Imerslund-Gräsbeck syndrome (I-GS), who have *CUBN* mutations that affect the C-terminal half of the protein, exhibit isolated proteinuria that has been interpreted as being due to defects restricted to albumin reabsorption by proximal tubular cells (Bedin et al., 2020). Without disregarding this interpretation, we consider that the data presented in this and in other studies do not exclude a potential role for podocyte CUBN impairment in the development of that disease. Indeed, there is evidence of glomerular hyperfiltration in some I-GS patients that might be consistent with increased albumin filtration (Bedin et al., 2020; Hauck et al., 2008; Storm et al., 2013). Furthermore, some studies report association of CUBAM mutations in humans with eventual loss of kidney function, consistent with a role for CUBAM in SD recycling under stress conditions (Ahluwalia et al., 2019; Bedin et al., 2020; Böger et al., 2011; Reznichenko et al., 2012). And finally, although not analysed in all studies, in I-GS patients there is evidence of increased urinary presence of proteins larger than albumin (e.g. transferrin, IgG) that may not be fully explained exclusively by decreased tubular reabsorption, and whose presence in the primary filtrate could be consistent with mild SD dysfunction (Storm et al., 2013; Wahlstedt-Fröberg et al., 2003). Therefore, determination of whether vertebrate CUBAM is also involved in SD recycling awaits further investigation.

## MATERIALS AND METHODS

### *Drosophila* strains

The following fly strains were used in this work: wild-type *Oregon R*, *Cubn2^E3-1^*, *Cubn^B2^*, *Amnionless^3^*, *Amnionless^NSI-1^* and *BAC^CH322-175013^* (this study, see below); *Idgf2-GFP* (Broz et al., 2017); *sqh-EYFP-ER* [Bloomington *Drosophila* Stock Center (BDSC), # 7195]; *dor^8^* (BDSC, # 28); *Df(2L)lt45, PPO1^Bc^/SM1, lt^16^* (BDSC, # 26187); *P{XP)-d06015* (*mgl^XP06105^* Exelixis Collection, Harvard Medical School, EP line for Mgl overexpression); *UAS-Amnionless-V5*, *UAS-mini-Cubn-Myc*, *UAS-mini-Cubn2-HA* and *UAS-hAMN-Myc* (this study); *UAS-lacZ* (BDSC, #3956); *UAS-GFP-Rab5* (BDSC, #43336); *UAS-RNAi-Cubn2* [Vienna *Drosophila* Resource Center (VDRC), #107024]; *UAS-RNAi-Rab5* (VDRC, # 103945); *UAS-RNAi-Rab1* (VDRC, #330620); *UAS-RNAi-Chc* (VDRC, # 103383); *UAS-RNAi-Sec6* (BDSC, # 27314); *UAS-RNAi-Sec15* (BDSC, # 27499); *prospero-GAL4* (a gift from C. Doe, HHMI, University of Oregon, Eugene, OR, USA); *sns-GCN-GAL4* (Zhuang et al., 2009); and *AB1-GAL4* (BDSC, # 1824). Unless otherwise specified, flies were maintained under standard conditions at 25°C.

### *CG42255* identification

*CG42255* (renamed *Cubn2*) was pinpointed in an RNAi candidate-based genetic screen aimed at identifying genes that participate in the formation or maintenance of the SD. 114 genes were selected either by their specific expression in embryonic nephrocytes (BDGP https://insitu.fruitfly.org/cgi-bin/ex/insitu.pl) or by their participation in pathways or processes presumed to be relevant for SD formation. *UAS-RNAi* lines obtained for these genes were crossed with the nephrocyte drivers *sns-GCN-GAL4* and/or *prospero-GAL4*, and their attenuation phenotypes evaluated by immunostaining with anti-Duf and anti-Pyd antibodies.

### Antibodies

The following antibodies were used: guinea-pig anti-Duf extracellular (1:100) and rabbit anti-Duf extracellular (1:400) (Weavers et al., 2009), rabbit anti-Pyd (1:100) and rat anti-PydEx5 (1:200) (Carrasco-Rando et al., 2019), rabbit anti-GFP (1:300) (ThermoFisher, A-6455), rabbit Cy3-anti-HRP (1:100) (Jackson ImmunoResearch, AB_2340262), mouse anti-V5 (1:500) (ThermoFisher, R960-25), mouse anti-cMYC 9E-10 (1:50 from DSHB; 1:1000 from BAbCO, 1244636002), rat anti-HA High Affinity (1:500) (Merk ROAHAHA Roche, 11867423001), guinea-pig anti-Boca (1:200) (Culi and Mann, 2003), guinea-pig anti-Sec15 (1:100) (Mehta et al., 2005), guinea-pig anti-Amnionless (1.100) (Ivy et al., 2015), and guinea-pig anti-Cubn2 (1:200), rat anti-Cubn (1:400) and guinea-pig anti-Mgl (1:500) (this study, validated by lack of staining in loss-of-function individuals).

### Oligonucleotides

The following oligonucleotides were used to generate gRNAs vectors: Cubn2-gRNA-TOP, CTTCGGAAGCCCTTCCCTTGTGTC; Cubn2-gRNA-BOTTOM, AAACGACACAAGGGAAGGGCTTCC; Cubn-gRNA-TOP, CTTCGCAAGTGGACCCTGCGAGAATG; Cubn-gRNA-BOTTOM, AAACCATTCTCGCAGGGTCACTTGC; Amnionless-gRNA-TOP, CTTCAAAGGATCGCCCCTTCTCTG; Amnionless-gRNA-BOTTOM, AAACCAGAGAAGGGGCGATCCTTT; AmnionlessNSI-gRNA-TOP, CTTCGTGACGCCACCTCCAAAGAC; AmnionlessNSI-gRNA-BOTTOM, AAACGTCTTTGGAGGTGGCGTCAC. Additional oligonucleotides were: anti-Cubn2-5′, GAATTCGGCGACTCCGAATCATCTCC; anti-Cubn2-3′, GCGGCCGCCCACTGGGTGCGGATACG; anti-Cubn-5′, GCATATGATAATCGTTGATGAA; anti-Cubn-3′, CTAACCGTAGTTATAGGAGGC; anti-mgl-5′, CATATGCGCAAGCGACCATTTGG; anti-mgl-3′, GGATCCTCAGCACTCGGACTTATC; Cubn2-RT, GCGTATGATGAAAGGACATCGC; Cubn-RT, CGAAGCGGCATTCCGTGGAATC; Cubn2-Asp718-5′, GGTACCATGCTCATTAAGTCGCTA; Cubn2-HindIII-3′, AAGCTTGAATCGCTGATGGGAGCA; Cubn-RI-5′, GAATCCCAAAATGGAAGGAGCTG; Cubn-RI-3′, GAATCCGCTGAAGCGCGTCTCGCA; Amnionless-5′, GAATTCCGATGGGTTTGCATTGG; Amnionless-3′, CTCGAGTAGATGGTCTCCTCGTC; hAMN-RI-5′, CGAATTCAACATGGGCGTCCTGGGCC; hAMN-RI-3′, CGAATTCGGCCTCGGCCTCGGC (Sigma); Cubn2-5′, GATCCGCACCACTGGCAATGTC; and Cubn2-3′, GTGGCCTATAAGAGGACTTCTG.

### Clones

Clones used were: dAMN cDNA IP03221 (BDGP, FBcl0293106), hAMN cDNA (Pedersen et al., 2010) and BAC CH322-175O13 (http://bacpacresources.org//).

### Immunohistochemistry

Third instar larval nephrocytes were dissected and fixed either in 4% formaldehyde dissolved in PBS for 2 h at room temperature or by heating the sample in PBS at 95°C for 10 s. Tissues were permeabilised by incubation in PBS containing 0.3% Triton X-100 (PBT), blocked in PBT containing 1% BSA and incubated with primary and secondary antibodies using standard procedures. For extracellular immunostaining of non permeabilised nephrocytes, tissues were fixed in 4% formaldehyde. After extensive washes in PBS, tissues were blocked in S2 culture media containing 10% foetal bovine serum and incubated overnight at 4°C with the corresponding primary antibodies, washed in PBS, permeabilised in PBT, incubated with the secondary antibodies and DAPI, washed and mounted for observation.

### Confocal and electron microscopy

Confocal and transmission electron microscopy were carried out following standard techniques. Tannic acid impregnation assays were performed as described by Kosaka and Ikeda (1983) by immersion of the specimens in 1% tannic acid in 0.1 M cacodylate buffer (pH 7.2) before the dehydration and embedding in Epon 812 steps. Samples for transmission electron microscopy were observed under a Jem1010 (JEOL) instrument working at 80 kV equipped with a TVIPS camera or a JEM1400 Flash transmission electron microscope at 100 kV equipped with a Gatan camera at the CBMSO TEM facility. Clathrin-coated structures were quantified from 60 micrographs of larval wild-type, strain Oregon R and 100 micrographs of *Cubn2^E3-1^* nephrocytes. Clathrin-coated structures were identified visually and counted from a 3 µm deep region underlying the nephrocyte surface. Total area analysed was ∼993 and 1626 µm^2^. For FIB-SEM analysis sample preparation and image acquisition were performed at the EMBL (Heidelberg) Electron Microscopy Core Facility. Samples were prepared and embedded in Durcupan following the minimal resin embedding protocol described by Schieber et al., (2017), and were imaged using a Crossbeam 540 (Zeiss) instrument working at 1.5 kV using the software ATLAS 3D (Fibics) (Narayan and Subramaniam, 2015). The resulting images were registered and a representative volume of 2.5 µm×2.5 µm×7.5 µm cropped in Fiji for further analysis. Segmentation and 3D reconstruction of SD, LCh and cortical tubules was performed in 3D Slicer using thresholding algorithms and manual editing for LChs, and manual segmentation based on morphology for SDs and tubules. The SD density at the cortical region was calculated by measuring the area of a model generated from the region of plasma membrane in contact with the extracellular matrix and the length summation of all SD rows that intersect with that surface model. The area of the SD rows was calculated estimating a width of 100 nm, the summation length and a rectangular shape. Fluorescent preparations were imaged and analysed using LSM 710, LSM800 (Zeiss) and SpinSR10 spinning disk (Olympus) confocal microscopes and Fiji software.

### Antibody production

To generate the anti-Cubn2 polyclonal antibody, a DNA fragment encompassing sequences encoding Cubn2 CUB domains 4 to 6 (Fig. S1A) was PCR amplified from genomic DNA using oligonucleotides anti-Cubn2-5′ and anti-Cubn2-3′ (listed previously) and cloned in the expression vector pGEX-4T-1. For the anti-Cubn antibody, a DNA fragment encoding Cubn CUB domains 10-11 (Fig. S1A) was obtained by PCR amplification of genomic DNA using oligonucleotides anti-Cubn-5′ and anti-Cubn-3′, and cloned into pET-14b. The corresponding GST-tagged and His-tagged fusion peptides were used to inoculate two guinea-pigs or two rats, respectively, following standard protocols. To generate anti-Mgl polyclonal antibody, a 490 bp region corresponding to the Mgl intracellular tail was PCR amplified with oligonucleotides anti-mgl-5′ and anti-mgl-3′, which introduce NdeI and BamHI restriction sites. The fragment was cloned into the expression vector pET-14b, the recombinant His-tagged fusion protein expressed in *E. coli*, and purified under native conditions by immobilised metal affinity chromatography. The antibody was raised in guinea pigs.

### Generation of novel *Drosophila* lines

We used the CRISPR/Cas9 technology to generate novel *Cubn2*, *Cubn* and *Amnionless* alleles according to Kondo and Ueda (2013.) Primers used to generate the gRNAs vectors targeting the different genes (see above) were designed using the http://www.flyrnai.org/crispr/ website. The novel alleles used in this study have already been listed and schematics showing the lesions induced are shown in Fig.S1B-D.

Site-specific transgenesis was used to obtain the *UAS-mini-Cubn2-HA*, *UAS-mini-Cubn-Myc*, *UAS-Amnionless-V5* and *UAS-hAMN-Myc* lines (Bischof et al., 2007). To obtain pUASTattB-mini-Cubn2-3xHA and pUASTattB-mini-Cubn-6xMyc, partial *Cubn2* and *Cubn* cDNAs were generated by retrotranscription from *Drosophila* total RNA using primers Cubn2-RT and Cubn-RT. The partial cDNAs were used as templates to produce by PCR amplification DNA fragments encoding Cubn2[Met1-Phe501] flanked by Asp718 and HindIII restriction sites and Cubn[Met1-Ser507] flanked by EcoRI restriction sites using oligonucleotides Cubn2-Asp718-5′ and Cubn2-HindIII-3′ or Cubn-RI-5′ and Cubn-RI-3′, respectively. The mini-Cubn2 fragment was fused to a HindIII-XbaI fragment coding three copies of the HA tag in an intermediary plasmid and the resulting Asp718-XbaI fragment was transferred to pUASTattB. Similarly, the mini-Cubn fragment was cloned into pUASTattB-6xMyc plasmid, which contains an EcoRI-XbaI fragment harbouring a C-terminal six tandem copies of the Myc tag. To generate pUASTattB-Amnionless-V5, the Amnionless coding region lacking the STOP codon and flanked by EcoRI and XhoI restriction sites was PCR amplified from cDNA IP03221 using primers Amnionless-5′ and Amnionless-3′. The EcoRI-XhoI fragment was cloned into pAc5.1-V5-His vector (Invitrogen) in frame with a C-terminal V5 tag. The *Amnionless-V5* fragment was excised by digestion with EcoRI and AgeI, and transferred to pUASTattB plasmid through additional intermediary cloning steps. To obtain pUASTattB-hAMN-6xMyc, a hAMN cDNA was used to amplify by PCR a DNA fragment comprising the hAMN-coding region lacking the STOP codon and flanked by EcoRI sites, using primers hAMN-RI-5′ (which includes consensus Kozak sequences optimised for *Drosophila*) and hAMN-RI-3′. This fragment, through additional subcloning steps, was transferred to pUASTattB-6xMyc vector. PCR amplification fidelity was checked by sequencing. The line *BAC^CH322-175O13^* was obtained by site-specific transgenesis using DNA from the clone BAC CH322-175O13 (http://bacpacresources.org//). To obtain the *Cubn2-GAL4* line, a 0.9 kb fragment obtained by PCR amplification on genomic *Oregon R* DNA (primers Cubn2-5′ and Cubn2-3′) was used to drive the expression of GAL4 after injection into *yw* embryos by P-element-mediated transgenesis.

### Co-immunoprecipitations

*UAS-mini-Cubn2-HA*, *UAS-mini-Cubn-Myc* and *UAS-Amnionless-V5* were co-expressed in *Drosophila* salivary glands using the driver *AB1-GAL4*. 80 salivary glands were dissected from third instar larvae in PBS, and homogenised in 600 µl of lysis buffer [150 mM NaCl, 50 mM Tris-HCl (pH 7.5), 1 mM EDTA, 1% Triton X-100 and 1 mM DTT] containing protease inhibitors (1 mM PMSF, 1X cOmplete and EDTA-free Protease Inhibitor Cocktail, Sigma Aldrich). The homogenate was incubated for 20 min at 4°C to ensure complete cell lysis and insoluble material removed by centrifugation at 16,000 ***g*** for 30 min at 4°C. For the co-immunoprecipitation, 300 μl of the same lysate were incubated overnight at 4°C with 25 μl Dynabeads of Protein G (Invitrogen) previously conjugated to 10 μg of mouse anti-Myc (BAbCO) following the manufacturer instructions. As control, 300 μl of the same lysate were incubated with 25 μl of dynabeads conjugated to 10 μg of unspecific rabbit IgGs. After three washes in lysis buffer, proteins were eluted from the matrix by incubation at 95°C for 5 min in 20 µl Laemmli sample buffer. Samples corresponding to the input, not-bound and elution fractions were analysed by immunoblotting using mouse anti-Myc (1:1000, BAbCO), mouse anti-V5 (1:3000) and High Affinity rat anti-HA (1:2500). Secondary antibodies were horseradish peroxidase (HRP)-conjugated anti-mouse and anti-rat (1:10,000, Jackson 115035003 and 112035003, respectively). The membranes were incubated with ECL Prime Western Blotting Detection Reagent and imaged in an Amersham 600 (GE Healthcare Life Sciences). For the blot shown in Fig. 3E, the elution lanes (control and IP-myc-mini-Cubn) were loaded with 20 times the amount compared with the input lysates for the anti-HA and anti-V5 blots and with four times the amount for the anti-Myc blot. Similar results were obtained in two additional experiments.

### Dextran uptake assay

Nephrocyte dextran uptake capacity was assessed *ex vivo*. For the experiment shown in Fig. S2, third instar larval garland nephrocytes were dissected, incubated with Texas Red dextran (10,000 Da ThermoFisher, D1863) at a concentration of 0.5 mg/ml in PBS at 25°C for 35 s, washed, chased for 5 min at 25°C, washed on ice, fixed and mounted. The experiment shown in Fig. S6 had the following variations: dextran was dissolved in S2 culture media, the uptake phase was 4 min and the chase phase was reduced to 10 s. To quantitate uptake data, an intensity threshold was defined to segment dextran puncta from background signal. This unique threshold was applied to all images from both control and experiment. The area of the segmented dextran puncta, as well as total cell area were calculated for each cell and their ratio obtained. Fiji software was used for image processing.

### Phylogenetic analysis of Cubilin proteins

The sequence of *D. melanogaster* Cubn protein was used as query to identify homologous sequences in the protein reference sequences database using NCBI Blast. High scoring hits, with high query coverage indicative of true paralogues were sorted by taxonomy. Organisms with more than one cubilin paralogue were further examined to exclude possible redundant, mis-annotated or too divergent sequences. Forty-eight sequences selected from organisms of representative taxa were aligned using NCBI COBALT and a phylogenetic tree generated using fast minimum evolution algorithm. The tree shown in Fig. S4 was generated with the TreeGraph2 software.

## Supplementary Material

Supplementary information

Reviewer comments
