# Supplementary material for: Endocytosis mediated by an atypical CUBAM complex modulates slit diaphragm dynamics in nephrocytes
Source: Development. 2021 Nov 30;148(22):dev199894. doi: 10.1242/dev.199894 (PMC8710305; doi:10.1242/dev.199894)
Supplement: Supplementary information [file develop-148-199894-s1.pdf]

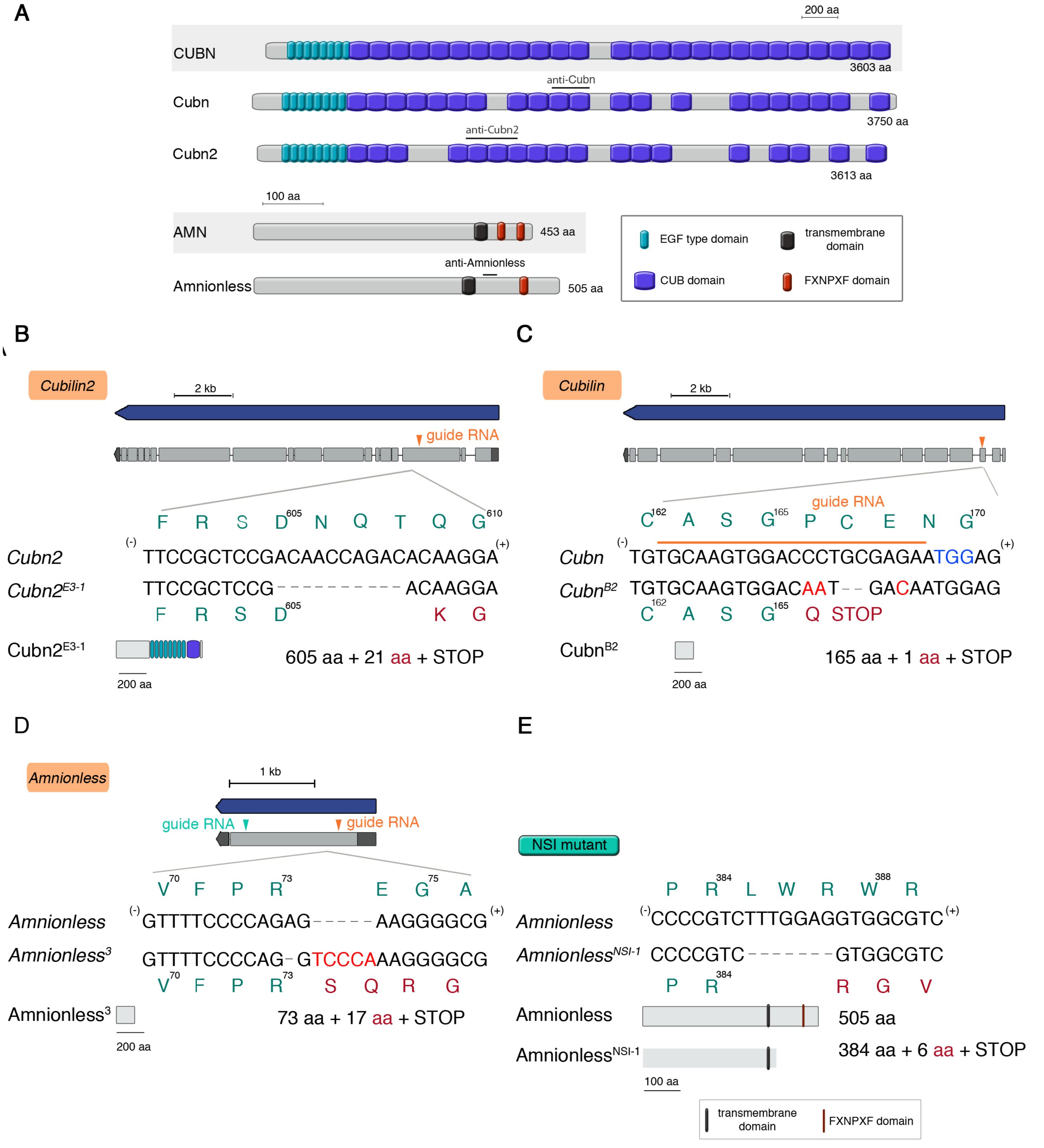

Fig. S1. Generation of novel mutants in CUBAM genes. (A) Domain composition of vertebrate (grey background) and Drosophila CUBAM members. Regions used for the generation of anti-Cubn and anti-Cubn2 polyclonal antibodies are indicated. (B-E) Schemes showing the selected regions for guide-RNA targeting (orange and green arrowheads above the transcripts depicted in grey) on *Cubn2* (B), *Cubn* (C) and *Amnionless* (D, E), the lesions generated on the DNA sequence and the resulting predicted truncated proteins, with mutant amino acids shown in red.

Development • Supplementary information

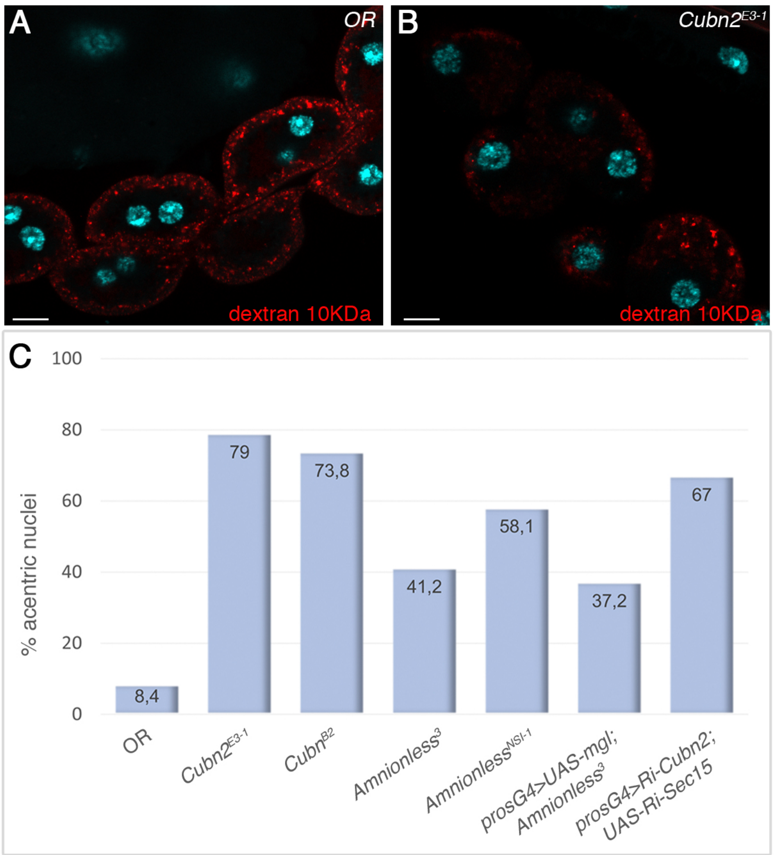

Fig. S2. Endocytic dextran uptake in *cubn-2*$^{E3\text{-}1}$ nephrocytes and quantification of acentric nuclei in CUBAM mutants. (A, B) Endocytic uptake of dextran (red) by nephrocytes is severely compromised in *cubn-2* mutants (B), compare with the wild-type (A). (C) Quantification of acentric nuclei present in larval garland nephrocytes of the indicated genotypes, represented as percentage of the total number of nuclei. *OR* (n=107), *Cubn2*$^{E3\text{-}1}$ (n=119), Cubn^{B2} (n=80), *Amnionless*^{3} (n=68), *Amnionless*$^{Nsi\text{-}1}$ (n=74), *p sG4>UAS-mgl; Amnionless*^{3} (n=156), *prosG4>UAS-Ri-Cubn2; UAS-Ri-sec15* (n=67). Scale bars: 10 µm.

Development • Supplementary information

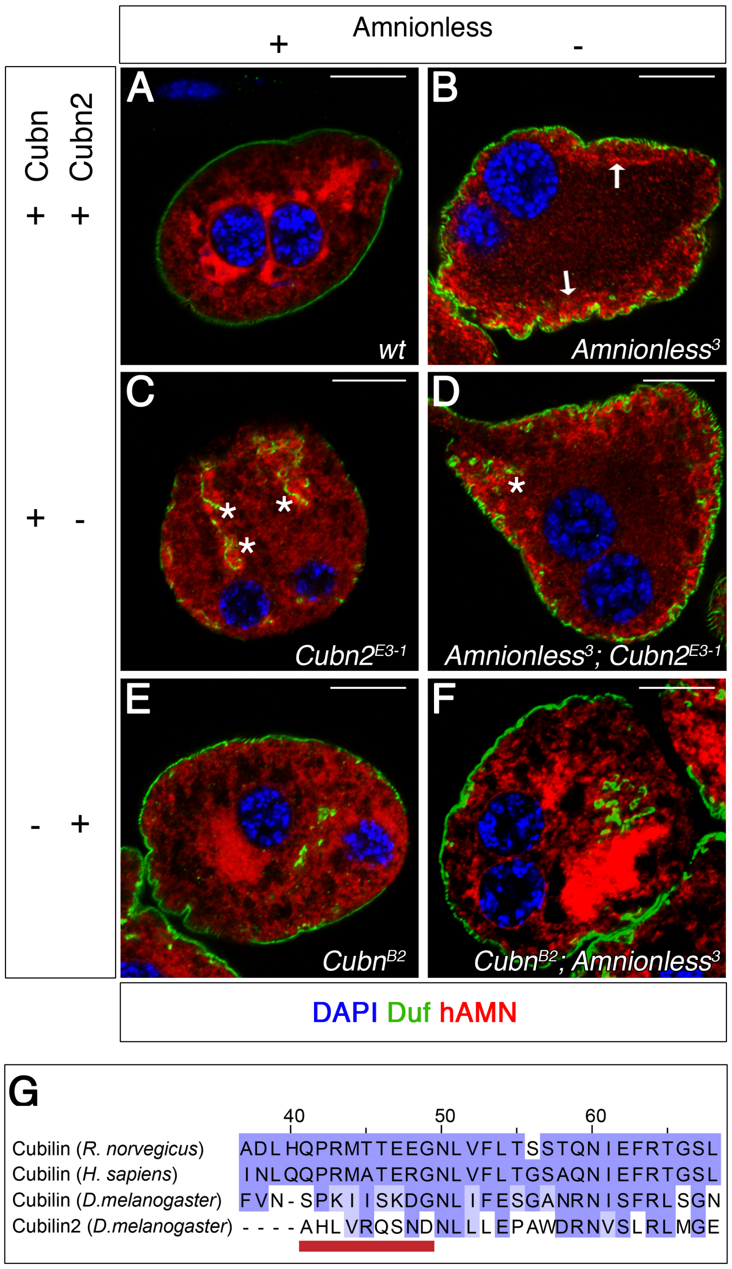

Fig. S3. Subcellular localisation of ectopic human AMN in wild-type and CUBAM mutant nephrocytes. (A,B) In a wild-type genetic background (Amnionless+, Cubn+, Cubn2+) hAMN overexpressed with *pros-G4* (red) is retained in the ER (A), whereas in the absence of endogenous Amnionless (B) it can traffic to the LCh membrane in the subcortical region (arrows). (C,D) In *Cubn2*$^{E3\text{-}1}$ mutants, hAMN traffics to the LCh membrane irrespectively of Amnionless presence. Asterisks point to the accumulation of hAMN close to ingressions of the external membrane labelled with anti-Duf. (E,F) In the absence of Cubn, hAMN is retained in the ER, even when there is no endoge-nous Amnionless (F). (G) There is amino acid conservation between vertebrate and *Drosophila* Cubn, but not with Cubn2, at the interface region of interaction with Amnionless (underlined in red). Similarities based on BLOSUM62 scores. Scale bars: 10μm.

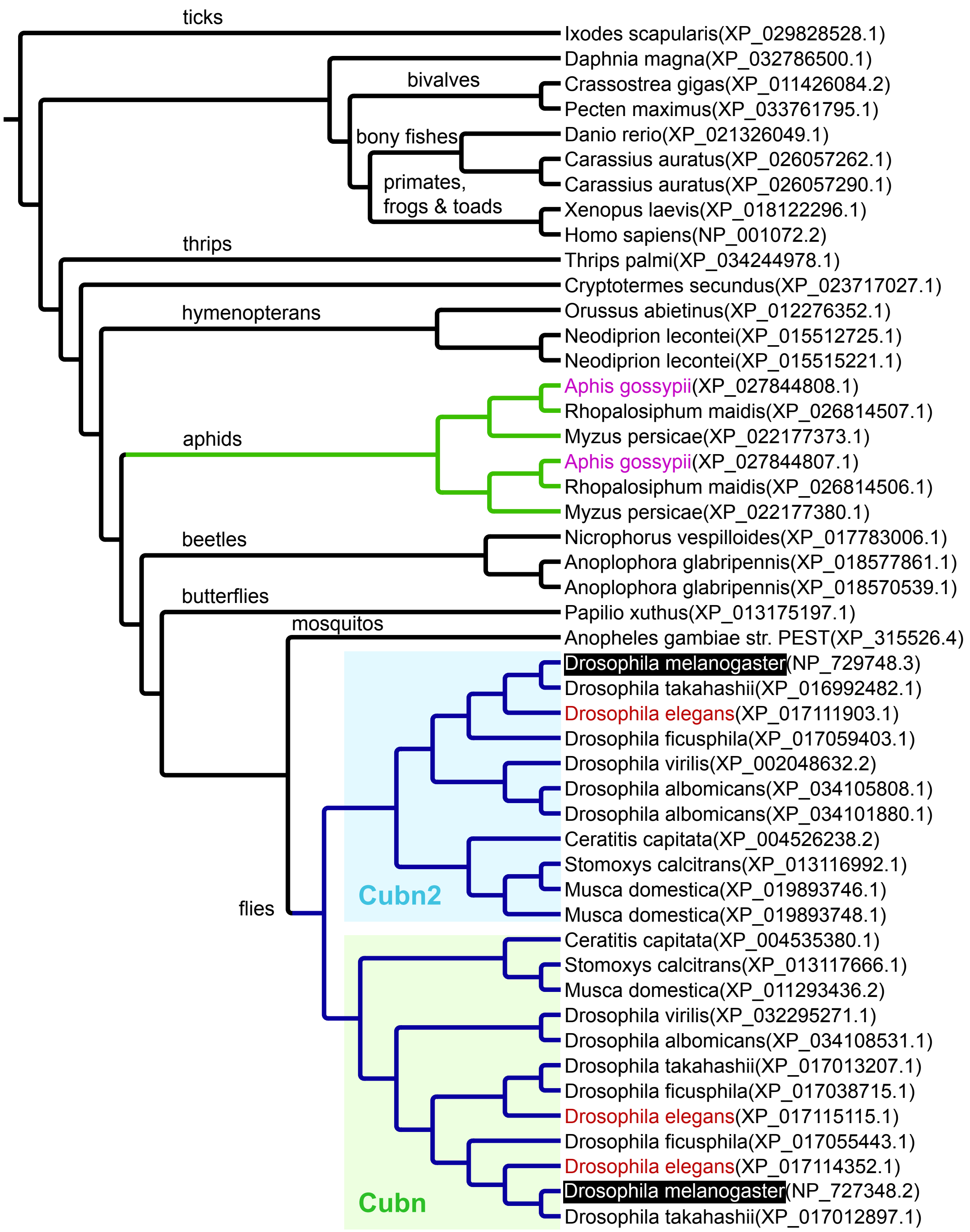

Fig. S4. Cladogram showing relationships of cubilin proteins from selected organisms. In flies there are two well-defined clusters of Cubilin paralogues, Cubn and Cubn2, highlighted with green and light blue backgrounds respectively. Interestingly, some fly species have three Cubilin paralogues resulting from a more recent duplication of cubn or of *Cubn2* genes. *Drosophila elegans* is highlighted in red as an example of a species with three Cubilin paralogues. A branch containing three aphid species is highlighted in green to point out that these species underwent a duplication of an ancestral cubilin gene similarly to flies. *Aphis gossypii* is highlighted in pink. Additional examples of cubilin duplications are also included in the cladogram.

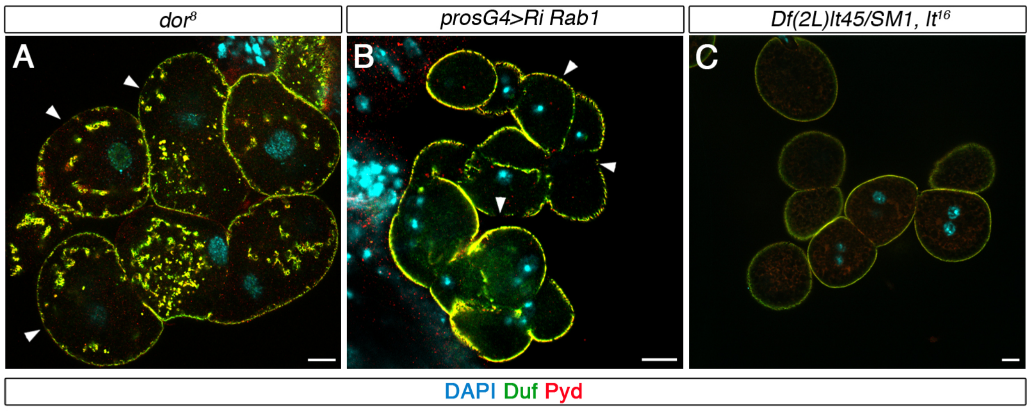

Fig. S5. SD positioning in several trafficking mutants. *dor8* mutants display deep ingressions of SDs similar to CUBAM LOF alleles (A). In contrast, depletion of *Rab1* (B) or *lt* (C) in nephrocytes does not induce internal accumulation of SD proteins. A decrease in the density of SDs can be observed in A and B (arrowheads). Compare with the wild-type in Fig.1A and CUBAM LOF mutants in 1B and 5E. Scale bars: 10µm.

Development • Supplementary information

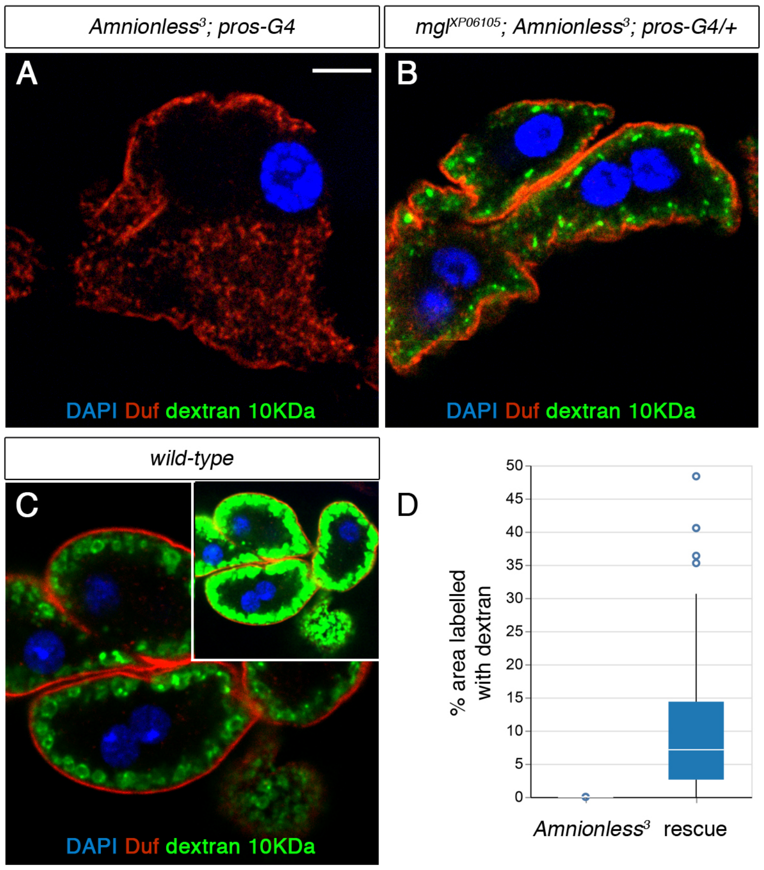

Fig. S6. Endocytic uptake of dextran in Mgl-rescued *Amnionless*^{3} nephrocytes. (A-C) 10 KDa ex vivo dextran uptake in the indicated genotypes (green). (A) *Amnionless*^{3}*; p s-GAL4* control nephrocytes show no dextran uptake.(B) Expression of Mgl (*mgl*$^{X 06105}$) with *p s-GAL4* increases uptake of 10 KDa dextran in *Amnionless*^{3} nephrocytes. (C) Dextran uptake in wild-type nephrocytes. The image display range for the dextran channel was optimized for better visualization. Inset shows the same image without optimization to compare with A,B. (D). Box-plot displaying quantification of dextran uptake (n=102N/16S). Scale bar: 10 µm, inset shown at a 50% reduction.

Development • Supplementary information

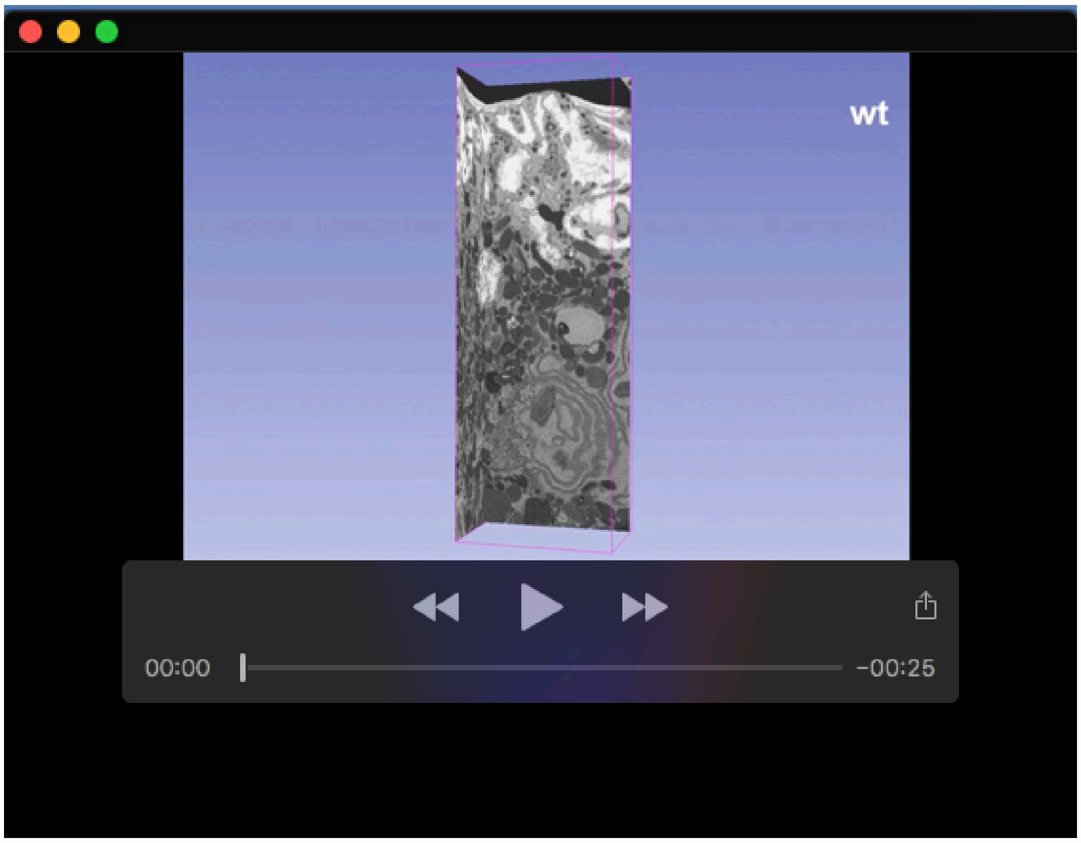

Movie l. Segmentation and 3D reconstructions of SDs, LCh and cortical tubules from wild-type and *Cubn2*$^{E3-1}$ larval garland nephrocytes, derived from FIB-SEM stacks.

Development • Supplementary information

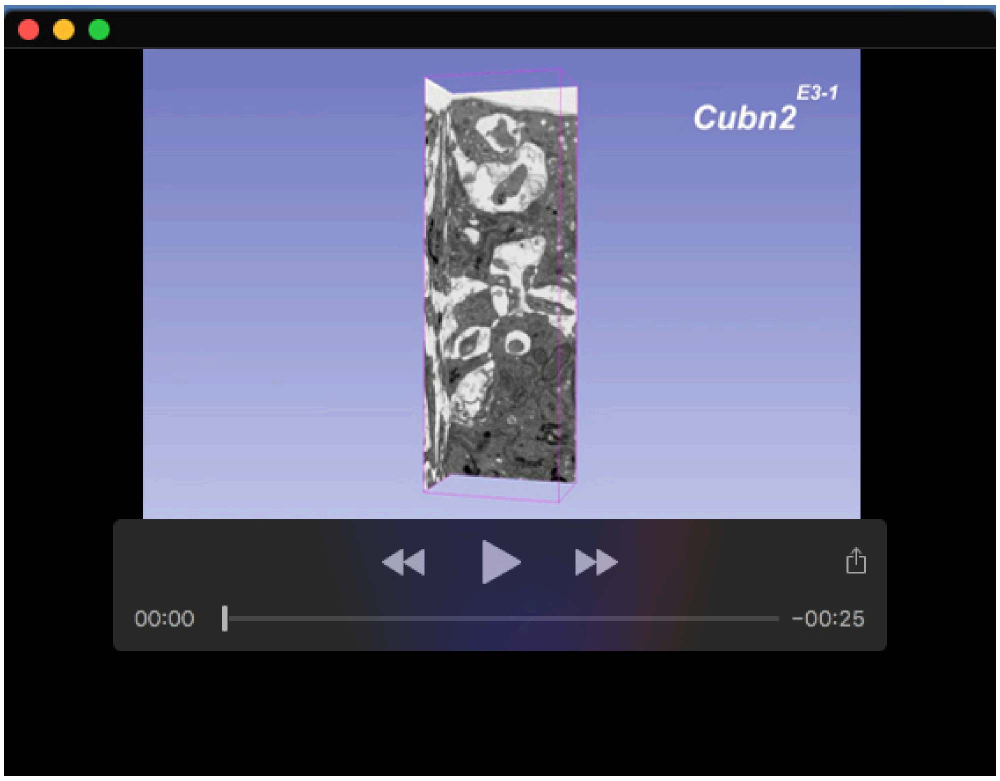

Movie 2. Segmentation and 3D reconstructions of SDs, LCh and cortical tubules from wild-type and *Cubn2*$^{E3\text{-}1}$ larval garland nephrocytes, derived from FIB-SEM stacks.

Development • Supplementary information
