# Supplementary material for: Endocytosis mediated by an atypical CUBAM complex modulates slit diaphragm dynamics in nephrocytes
Source: Development. 2021 Nov 30;148(22):dev199894. doi: 10.1242/dev.199894 (PMC8710305; doi:10.1242/dev.199894)
Supplement: Reviewer comments [file develop-148-199894_review_history.pdf]

Endocytosis mediated by an atypical CUBAM complex modulates slit diaphragm dynamics in nephrocytes

Alexandra Atienza-Manuel, Vicente Castillo-Mancho, Stefano De Renzis, Joaquim Culi and Mar Ruiz-Gómez

DOI: 10.1242/dev.199894

Editor: Cassandra Extavour

Review timeline

Original submission:	14 June 2021
Editorial decision:	23 July 2021
First revision received:	14 October 2021
Accepted:	22 October 2021

Original submission

First decision letter

MS ID#: DEVELOP/2021/199894

MS TITLE: Endocytosis mediated by an atypical CUBAM complex modulates slit diaphragm dynamics in nephrocytes.

AUTHORS: Alexandra Atienza-Manuel, Vicente Castillo-Mancho, Stefano De Renzis, Joaquim Culi, and Mar Ruiz-Gómez

I have now received all the referees reports on the above manuscript, and have reached a decision. The referees' comments are appended below, or you can access them online: please go to BenchPress and click on the 'Manuscripts with Decisions' queue in the Author Area.

The overall evaluation is positive and we would like to publish a revised manuscript in Development, provided that the referees' comments can be satisfactorily addressed. Please attend to all of the reviewers' comments in your revised manuscript and detail them in your point-by-point response. If you do not agree with any of their criticisms or suggestions explain clearly why this is so.

We are aware that you may currently be unable to access the lab to undertake experimental revisions. If it would be helpful, we encourage you to contact us to discuss your revision in greater detail. Please send us a point-by-point response indicating where you are able to address concerns raised (either experimentally or by changes to the text) and where you will not be able to do so within the normal timeframe of a revision. We will then provide further guidance. Please also note that we are happy to extend revision timeframes as necessary.

Reviewer 1

Advance summary and potential significance to field

Atienza-Manuel et al report on a new ortholog of Cubilin, Cubilin-2, which was found in flies when performing an uptake screen in nephrocytes. The authors nicely show that Cubilin-2 is incorporated into the Cubam complex, thereby promoting uptake of protein ligands. Absence of Cubilin-2 and

© 2021. Published by The Company of Biologists under the terms of the Creative Commons Attribution License (https://creativecommons.org/licenses/by/4.0/).

the other Cubam members leads to strongly reduced protein uptake and to misplaced slit diaphragms (SD). The latter finding is suprising as in mammals Cubam is mainly responsible for endocytosis in proximal tubular cells, whereas a function in podocytes that harbor SD has not been reported. Interestingly, SD mispositioning to invaginations can be rescued by inhibiting exocytosis or boosting endocytosis, suggesting that this phenotype stems from an imbalance in endo- and exocytosis.
Overall, the paper provides interesting insights and new tools for nephrocyte and Cubam biology. In my opinion, the relevance to the mammalian kidney is overstated, also because the authors fail to acknowledge the differences between nephrocytes and mammalian kidney. A big deficit throughout the paper is lack of quantifications.

Comments for the author

Major points:
1. p.5 It would be nice to know a little more about the screen in which Cubilin-2 was identified.
2. Why is the Duf staining in cubn-2 3-1 so different in Fig. 1C vs. Fig. 4D? In Fig. 4 central aggregates are counted, but the pictures do not shos them.
3 Why is the ER morphology different between cubnB2 and dAMN3 (Figure 3J,K vs.3L,M)?
4. In Fig. 6, functional effects (such as enhanced protein uptake) should be demonstrated for ectopic Mgl expression or Rab5 CA. Similarly, KD effects for sec15 on exocytosis should be addressed.
5. Quantifications are lacking in many figures. Especially for the TEM, this is absolutely necessary.
6. Some phenotypes like ER expansion and multilamellar bodies are not at all explained.
7. Newer single cell RNA-seq studies (PMID: 31689386; PMID:29622724) do not support Cubilin expression in podocytes. The cited papers that show Cubam expression in podocytes are problematic because they rely on cultured podocytes (Gianesello) or antibody stainings without antibody validation in knockout animals (Prabakaran). Neither CUBN mutations in humans (PMID: 31613795) nor conditional CUBN KO in mice (PMID: 20798259) support podocyte/SD phenotypes such as glomerular proteinuria. Thus, the central point in the paper - role of Cubam in SD endocytosis - may not be relevant for the mammalian kidney. This needs to be at least discussed.

Minor points:
- There is generally very little information on nephrocyte dissections in the methods. What are, for example mature nephrocytes (line 157, p.6)?

Reviewer 2

Advance summary and potential significance to field

This study by Atienza-Manuel et al. reported an interesting discovery about an atypical CUBAM complex in regulating receptor-mediated endocytosis and the slit diaphragm dynamics in Drosophila nephrocytes. They identified Cubn-2, a paralogue of Cubn, in forming a tripartite complex with Amn and Cubn. Interestingly, this CUBAM receptor complex is required for nephrocyte slit diaphragm dynamics.
Mutations in the CUBAM complex led to ingressions of external membrane and slit diaphragm mislocalization. They proposed these phenotypes were caused by an imbalance between endocytosis and exocytosis in these cells, as suppressing exocytosis or increase endocytosis could partially restore slit diaphragm-positioning in CUBAM mutants. In summary, the identification of Cubn-2 and the organization of the atypical CUBAM complex, as well as the finding that the CUBAM complex is important for SD protein recycling is interesting. However, the mechanism of how CUBAM complex regulate SD dynamics is confusing and needs further clarification and more evidence are needed to support the model proposed in the last figure. The potential significance to the field is questionable since no podocyte defects have been reported in animal models of cubilin deficiency.

Comments for the author

Major concerns and comments:

© 2021. Published by The Company of Biologists under the terms of the Creative Commons Attribution License (https://creativecommons.org/licenses/by/4.0/).

1) As the authors mentioned, recent studies have found that Cubulin and Amn are expressed in murine and human glomerular podocytes. It is reasonable to assume that the CUBAM-mediated endocytosis may also regulate slit diaphragm dynamics in mammalian podocytes, as it did in Drosophila nephrocytes. However, no consistent glomerular podocyte pathology has been found in human patients with Imerslund-Grasbeck syndrome or animal models of cubilin deficiency. This argues against a major role of CUBAM complex in regulating slit diaphragm dynamics in mammalian podocytes.

2) The model in Figure 7 showed that slit diaphragm proteins are directly endocytosed by the CUBAM-mediated endocytosis. This speculation needs more evidence, since the authors did not show any interaction between slit diaphragm proteins and CUBAM complex, colocalization between these proteins, or other direct evidence.

3) Some results in this paper (and from literatures) could be interpreted against CUBAM-mediated endocytosis of slit diaphragm proteins. For example, the phenotypes of knocking-down genes in the clathrin pathway and major components of endocytic pathway appear different from that of CUBAM mutants. The phenotype of chc and Rab5 knockdown (Figure 5D and 5E) exhibited clear differences in slit diaphragm localization from CUBAM mutants. If the endocytosis of slit diaphragm proteins were mainly mediated by CUBAM complex, shouldn't we see similar defects?

4) The results of partial rescue of slit diaphragm ingressions by silencing sec6 or sec15, overexpression of activated Rab5 or megalin in CUBAM mutant nephrocytes are confusing. These genetic manipulations all partially rescued slit diaphragm ingressions of CUBAM mutants, yet each showed different slit diaphragm mislocalizations on cell membrane. If the phenotypes in CUBAM mutants were due to imbalance between endocytosis and exocytosis, why only the ingressions were rescued? What is the reason for the phenotypic differences among these genetic manipulations?

5) In Panel A of the model, the authors hypothesize that CUBAM facilitates the SD components degradation or recycling through a cortical tubular network. However there is no direct evidence to demonstrate this. To claim that "the main function of the nephrocyte cortical tubular network is to quick recycling of receptor-ligand complexes", more evidence is needed. Maybe the authors could consider genetically alter the nephrocyte cortical tubular network and examine its effect on the recycling of receptor-ligand complexes.

6) In Panel B of the model, it is confusing why “a reduction in the recycling of SD components” can lead to accumulation of extra SDs at subapical locations and in deep regions of the enlarged labyrinthine channels. If SD recycling is reduced as shown in the model, shouldn’t the number of SD be reduced as well? If extra SD were found in CUBAM mutants, wouldn't it suggest an increased SD recycling or an increased production of new SD proteins?

7) The discussion for the model is confusing. In Page 19, line 510, it says
“possibly by promoting the endocytosis of SD components for its posterior degradation and/or recycling (Fig. 7)”. What does “posterior degradation and/or recycling” mean?

Reviewer 3

Advance summary and potential significance to field

In this manuscript, the authors characterize a Drosophila-specific paralog of Cubn (which they named Cubn-2) that is required for an atypical CUBAM complex-mediated endocytosis (CUBAM has a known role in the vertebrate renal proximal tubule) and proper slit diaphragm distribution in fly nephrocytes. Since the CUBAM complex is also expressed in vertebrate podocytes, this suggests that it contributes to slit diaphragm maintenance in vertebrates, too.

Comments for the author

The manuscript presents solid and good-quality experimental data, and I think that submitting to Development was a good choice. Still, the manuscript could be and should be strengthened before publication (see below).

© 2021. Published by The Company of Biologists under the terms of the Creative Commons Attribution License (https://creativecommons.org/licenses/by/4.0/).

Major comments:
1. I don't think that the claim of Idgf2 being an endogenous ligand for this endocytic receptor complex (line 155) is properly supported by the data, as the effect could also be indirect. Please tone down this statement if you can't provide e.g. biochemical binding data.

2. The conclusion of decreased endocytosis in mutants is based on Idgf2-GFP dextran uptake and TEM experiments, the latter without quantification. It would be useful to expand these data by visualizing and quantifying clathrin Rab5 and Rab7 structures in different genotypes using confocal microscopy.

Minor comments:
3. Representative images should be included for Fig 4i (e.g. as supplemental material)
4. Do Fig 6h-j also show tannic acid impregnation TEM? Why are invaginations colored light blue in panel i? Why are black arrowheads pointing to the plasma membrane in panel j? Please clarify these.

First revision

Author response to reviewers' comments

Detailed list of responses to the reviewers: Reviewer #1

Major points:

1. p.5 It would be nice to know a little more about the screen in which Cubilin-2 was identified.

Authors' response:
Following the reviewer's suggestion, in the revised version of the manuscript we included a new heading in the M&M section (line 513) describing the RNAi genetic screen that identified *Cubn-2*.

2. Why is the Duf staining in cubn-2 3-1 so different in Fig. 1C vs. Fig. 4D? In Fig. 4 central aggregates are counted, but the pictures do not shows them.

Authors' response:
There are two main reasons for the differences in Duf staining noticed by the reviewer. First, *cubn-2*$^{E3-1}$ phenotype regarding Duf distribution ranges from ingressions near the cell periphery to stronger phenotypes displaying Duf central aggregates as shown in Figure 1C. In this regard, the range of *cubn-2*$^{E3-1}$ phenotypes is similar to that of *dAMN*^{3}, which we quantify in Figure 4I. Our main aim in Figure 4A-G' was to show the changes in the extracellular distribution of Cubn and Cubn-2 in different genetic backgrounds.
Thus, we selected the most simple examples that illustrate those changes, with nephrocytes displaying few membrane ingressions, as the nephrocyte we showed in panel 4D in the previous manuscript version. However, we agree that this may lead to some confusion, so we have substituted the panel 4D for a similar nephrocyte but with a stronger ingressions phenotype, imaged in the same experiment. This does not change in any way the interpretation of the results shown in Figure 4.
A second reason that contributes to apparent weaker phenotypes of Duf ingressions in Figure 4A-G' is that the figure shows the extracellular distribution of Cubn, Cubn-2 and Duf. In this protocol, we incubate non-permeabilized tissue with the corresponding primary antibodies. We have noticed that using this protocol, it is hard for the antibodies to reach the more internal regions of the labyrinthine channels and thus, Duf central aggregates are not always detected.
Finally, following suggestions made by reviewers 1 and 3, in the revised version of the manuscript we include examples of the phenotypic classes used for Figure 4I quantification.

3 Why is the ER morphology different between cubnB2 and dAMN3 (Figure 3J,K vs.3L,M)?

© 2021. Published by The Company of Biologists under the terms of the Creative Commons Attribution License (https://creativecommons.org/licenses/by/4.0/).

Authors' response:
In the manuscript, we show that in *cubn*^{B2}, *dAMN*^{3} or *cubn-2*$^{E3-1}$ mutants, the CUBAM complex cannot assemble correctly in the ER, possibly resulting in the accumulation of misfolded monomers or aberrantly assembled CUBAM complexes in this organelle. This could trigger the ER unfolded protein response (UPR), since hydrophobic protein surfaces would be exposed. It was described that UPR might alter lipid homeostasis and induce an expansion of ER membranes, causing changes in ER morphology. This could explain the enlarged ER observed in *dAMN*^{3} mutants. However, analysing why the ER is bigger in *dAMN*^{3} compared to *cubn*^{B2} or *cubn-2*$^{E3-1}$ mutants that show a more dispersed ER morphology, would require the detailed examination of the UPR in the different CUBAM mutant backgrounds and their effect on lipid synthesis, which, albeit interesting, it is clearly outside the scope on this manuscript.

4. In Fig. 6, functional effects (such as enhanced protein uptake) should be demonstrated for ectopic Mgl expression or Rab5 CA. Similarly, KD effects for sec15 on exocytosis should be addressed.

Authors' response:
Regarding the first part of the comment, we agree with the reviewer and in the revised version of the manuscript we include a new Supplementary Figure (Fig. S6) showing that Mgl overexpression in a *dAMN*^{3} background increases dextran uptake.
As for the second point, to demonstrate that the knock-down of exocyst subunits results in a reduction of exocytosis in nephrocytes would be technically very challenging.
Nephrocytes are highly secretory cells. Accordingly, our unpublished RNAseq analyses indicate they express high levels of multiple secreted proteins. However, these are mostly small peptides of possible immune function or of unknown function for which no antibodies are available, making it difficult to track these proteins through the secretory pathway. However, we are confident that our RNAi lines are blocking the exocyst complex as expected. In this regard, a recent paper from Dr. Han's lab (PMID:32238475) showed that sec15 silencing results not only in a reduction of Sec15 but also of other exocyst proteins in nephrocytes, suggesting a global impairment of exocyst function. In addition, they also showed that SD density in the surface of the nephrocytes is reduced after blocking the exocyst, a phenotype that we also observe in our knockdowns.

5. Quantifications are lacking in many figures. Especially for the TEM, this is absolutely necessary.

Authors' response:
We understand the reviewer's concern and in the revised version of the manuscript, we made an effort to quantitate additional data.

First, following the reviewer's suggestion we have included new data showing that ectopic expression of Mgl in *dAMN*^{3} nephrocytes increases endocytosis. Quantification in Figure S6 was made from a total of 102 nephrocytes from 16 different larvae as described in the M&M section and in the figure legend.

Second, our statement that *cubn-2*$^{E3-1}$ mutants have reduced endocytosis is supported by the quantification of the uptake of Idgf2-GFP, presented in Figure 1G of the previous manuscript version, and now also complemented by the quantification of clathrin-coated structures from TEM micrographs in the wild-type (n=60) and in *cubn-2*$^{E3-1}$ (n=100) mutants, which shows a dramatic reduction (10 times) of these structures in the 3µm subcortical region of cytoplasm beneath the plasma membrane. The reason why this quantification was not included in the previous version of the manuscript is the distinct morphologies of the subcortical region in the wild-type and in *cubn-2*$^{E3-1}$ mutants, which precludes a precise comparison of the data obtained. Thus, considering a depth of 3µm of the subcortical region, as we did in our quantification, labyrinthine channels occupy an important part of it in the wild-type, while the region free of channels is larger in the mutant, which may lead to an underestimation of the density of clathrin-coated elements in the wild-type. See figure 1A-D (showing representative images) and micrographs shown below.

© 2021. Published by The Company of Biologists under the terms of the Creative Commons Attribution License (https://creativecommons.org/licenses/by/4.0/).

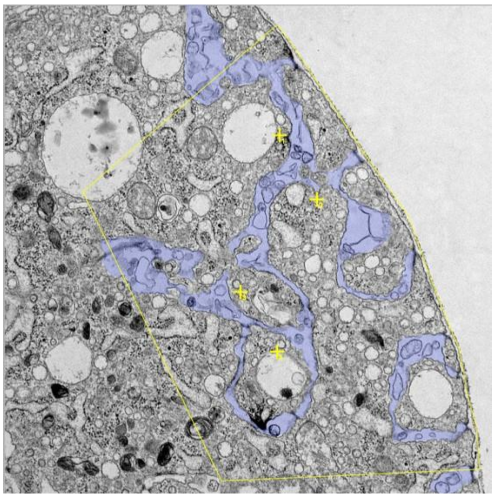

cubn-2^{E3-1}

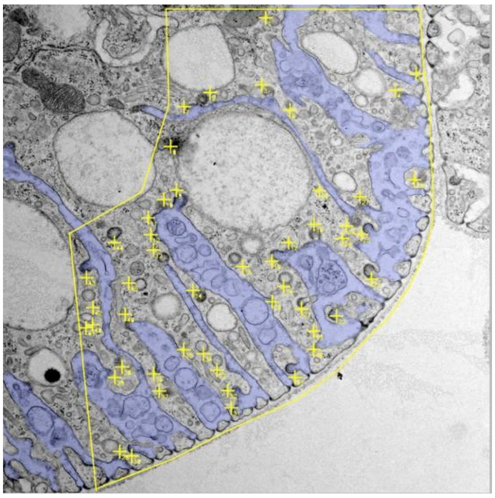

wild-type

And third, the reduction in the density of surface SDs in *cubn-2*$^{E3-1}$ mutants, shown in the previous version of the manuscript by the analysis of confocal microscopy images, for example Figure 1A and B, is now also supported by measurements performed in our 3D models obtained by FIB-SEM. We believe the use of a 3D model for this type of analysis is critical, since single plane TEM images cannot capture whether the parallel rows of SDs were sectioned perpendicular or at an angle, which would result in different measurements for identical 3D structures. Our measurements of the 3D models indicate that in the wild-type, a 35% of the nephrocyte surface is covered by rows of SDs. This decreases to 16% in the *cubn-2*$^{E3-1}$ mutants, as it is now included in figure 2.

TEM micrographs shown in Figure 6 were removed in the revised version. We agree with the reviewer that in the absence of accurate data quantitation these micrographs were not too relevant.

6. Some phenotypes like ER expansion and multilamellar bodies are not at all explained.

Authors' response:
Even though we agree that the ER expansion phenotypes and the appearance of multilamellar bodies are interesting observations, they are not the focus of our paper. Accumulation of misfolded proteins in the ER results in the activation of the unfolded protein response, which can regulate lipid homeostasis and in some cases, was reported to result in the expansion of ER membranes. When one of the components of the CUBAM complex is absent, we observe and describe in the manuscript an accumulation of the other components in the ER, an effect also described in the mammalian CUBAM literature. This could explain the ER enlargement we observe in mutations in CUBAM components. However, taking into account space limitations, we consider that the discussion of these possibilities in our manuscript is not necessary. Regarding the multilamellar bodies, we discuss they were described to be associated with defects in endocytosis (Dermaut et al., 2005), which could explain their appearance in several of our experimental manipulations.

7. Newer single cell RNA-seq studies (PMID: 31689386; PMID:29622724) do not support Cubilin expression in podocytes. The cited papers that show Cubam expression in podocytes are problematic because they rely on cultured podocytes (Gianesello) or antibody stainings without antibody validation in knockout animals (Prabakaran).
Neither CUBN mutations in humans (PMID: 31613795) nor conditional CUBN KO in mice (PMID:20798259) support podocyte/SD phenotypes such as glomerular proteinuria. Thus, the central point in the paper - role of Cubam in SD endocytosis - may not be relevant for the mammalian kidney. This needs to be at least discussed.

Authors' response:
The reviewer questions published human CUBN and AMN expression data in podocytes because they

© 2021. Published by The Company of Biologists under the terms of the Creative Commons Attribution License (https://creativecommons.org/licenses/by/4.0/).

were obtained in cultured podocytes or using antibodies not validated in KO animals. Nonetheless, the specificity of the antibody used in the Prabakaran article was not considered problematic in other studies (PMID:24156255; PMID:25052491), one of which was accepted to demonstrate CUBN expression in the human ileum. However, we agree with the referee that convincingly showing that a specific gene or protein is expressed in a specific human cell type may be challenging, especially if the amount of mRNA or protein is low. However, we respectfully disagree with the referee regarding podocyte expression of *CUBN* and *AMN* in mice and humans in single cell studies. On the one hand, data from one of the papers the reviewer quoted (PMID:29622724), indicate that both *Cubn* and *Amn* are expressed in mice podocytes. Thus, in Table S3, presenting the gene expression data for specific genes in all cell groups described in that study, the % of cells in the podocyte group (Group 2) expressing *Cubn* or *Amn* (8,974% or 7,692%) is about one third of the % of proximal tubular cells (Group 3) expressing these genes (30,715% or 24,194%), whereas the % of cells expressing the SD genes *Nphs1* or *Tjp1* in proximal tubular cells is residual in the case of *Nphs1* (0,060%) and 16- fold lower compared to podocytes for *Tjp1* (2,122%). On the other hand, in the revised version of the manuscript we now refer to data from Humphries laboratory (http://humphreyslab.com/SingleCell/) that clearly demonstrate *CUBN* and *AMN* gene expression in human podocytes. In fact, podocytes are the cells with higher *CUBN* and *AMN* expression in the nephron after proximal tubular cells, while mRNA for both is absent from mesangial cells.

In addition, the referee raises an interesting point regarding the human phenotype of *CUBN* mutations, a concern also manifested by reviewer 2. We agree with the reviewers that this phenotype has not been fully characterised. However, there is some recent information that may shed light into the potential contribution of podocyte CUBN to normal kidney function. In the first place we would like to consider the phenotypes we described in *Drosophila*, where mutations in CUBAM components result in lower density of SDs, but maintain still a fair number of diaphragms. Based on this, we would not expect a severe podocyte phenotype in humans if CUBAM played a similar role, but it could be possible that the phenotype become evident under stress conditions. Revising published data on Imerslund-Grasbeck syndrome (I-GS) patients we find that:

1. *CUBN* mutations may result in I-GS, in which the main phenotype is vitamin B12 deficiency, or in isolated proteinuria. Patients with isolated proteinuria have mutations affecting specific CUBN domains.
2. Currently, it is thought that patients with *CUBN* mutations resulting in isolated proteinuria have defects restricted to albumin reabsorption by proximal tubular cells. Indeed, Bedin et al., 2020 (PMID:31613795) recently described a large cohort of patients (n=39) presenting chronic isolated proteinuria and early childhood onset, associated with biallelic pathogenic CUBN variants. They concluded that “there is an important role for the C-terminal half of CUBN in renal albumin reabsorption and that albuminuria due to reduced cubulin function could be an unexpectedly common benign condition in humans that may not require any proteinuria-lowering treatment or renal biopsy”.

While in principle we agree with this interpretation of the data, we argue against considering that they exclude a role of podocyte CUBN. In this regard, we would respectfully argue that science remains open to alternative interpretation of data once new knowledge becomes available, as it is the case for our data on CUBAM in *Drosophila*. Thus, we would like the reviewers to consider the following arguments that potentially may open the door to further research along the lines identified by our *Drosophila* work, i.e., while not proving the clinical relevance of the *Drosophila* observation, they are at least consistent with such potential clinical relevance:

I. The Bedin cohort is mostly young, with a mean age at last follow-up of 10 to 26 years (table 1). This precludes conclusions on the long-term outcome of kidney function, as stated by the authors: “Although adverse long-term effects associated with *CUBN* deficiency cannot be fully excluded”.
II. While Bedin figure 4.D shows that renal function of patients with biallelic filtered CUBN variants (n = 35) was found to be declining according to normal aging, older patients are clearly underrepresented as compared to the comparator cohort. However, supplementary figure 1.D showing eGFR values taken at different disease progression times, discloses that the tree patients aged more than 35 had a dramatic decrease in eGFR over a short period of time. There are multiple potential explanations for this observation. However, the data are also consistent with a SD recycling defect that may become relevant upon stress conditions, leading to rapid loss of kidney function. In this regard, CUBN variants associated

© 2021. Published by The Company of Biologists under the terms of the Creative Commons Attribution License (https://creativecommons.org/licenses/by/4.0/).

with albuminuria had a greater impact on albuminuria in diabetic kidney disease, a condition in which albuminuria is associated with progressive loss of kidney function, than in controls (PMID: 30547231, PMID: 21355061). Furthermore, CUBN variants associated with proteinuria were also associated with renal function loss in two independent settings: ESRD in native kidneys and in transplanted kidneys (PMID: 22574174). This would not be predicted nor explained by an exclusive tubular role of cubilin in albuminuria.

III. They also say that "although not measured in all patients, the proportion of albumin in the urinary protein was higher than 50%, and urinary α1- or β2- microglobulin was mostly low or absent". We agree with Bedin et al., 2020 that this argues against a generalized tubular defect in low molecular weight protein reabsorption and differs from other forms of tubular proteinuria, including megalin deficiency. However, these data may also be consistent with a mild increase in albumin (MW 66 KDa) filtration. And, as Bedin et al describe, this may not be deleterious as albuminuria has several adverse effects that depend on albumin uptake by tubular cells that will not take place in patients with mutations in *CUBN*. Then a relevant question now becomes, what about other larger proteins such as transferrin (MW 80 KDa), or IgG (MW 150 KDa) that are typically filtered in excess when the filtration barrier is not fully functional? Bedin et al do not provide data on this. However urinary protein was increased (mean 0.69 to 1.0 g/day), albumin accounted for around 60% of urinary protein and low molecular weight proteins were low or absent. Thus, other, likely larger, proteins contributed from mean 200 to mean 400 mg/day non-albumin proteins in urine in these patients. Therefore, to our knowledge this has not been explored in depth for patients carrying only-proteinuric CUBN variants. However, Wahlstedt-Fröberg et al (PMID: 12687456) analysed proteinuria in cubilin-deficient patients with selective vitamin B12 malabsorption. They reported that urinary transferrin was below the detection limit in controls and in 6/7 patients with mutations in *CUBN* with low proteinuria and low albuminuria, but was increased (up to 18-fold) in 6/6 patients with higher albuminuria. IgG was measured with a sensitive assay in only 2 controls (0.2mg/mmol creatinine) and 3 patients (0.3-0,8 mg/mmol, i.e., from 50% to 4-fold higher than in controls). In another study of proximal tubular function in I-GS, increased urinary excretion of transferrin was also reported (PMID: 24156255), and further reports also found increased urinary transferrin (1.17mg/mmol creatinine; normal<0.2) and IgG (0.98mg/mmol creatinine; normal<0.2) (PMID: 17668238). While it may be argued that these are also cubilin ligands, and their presence in urine may might be due to insufficient proximal tubular reabsorption, their larger size means that their presence in urine may also reflect and be compatible with higher filtration. Indeed, cubilin-independent transferrin tubular reabsorption was observed in mice (PMID: 20798259).

IV. The eGFR values reported by Bedin et al for their cohort were normal or even in the high normal range for younger patients (95% CI up to 172 ml/min/1,73m^2), but additionally four C-terminal missense CUBN variants were associated with albuminuria and an increased eGRF in population-based studies. Excessive filtration of albumin may result in higher eGFR as it may increase the oncotic pressure in the urinary side of the glomerular filtration barrier. Thus, these data may also be consistent with a mild increase in albumin filtration. Similarly, previous reports of I-GS patients also describe low serum creatinine values that, among other possibilities, may be consistent with glomerular hyperfiltration (PMID: 24156255), or confirmed hyperfiltration by increased creatinine clearance (PMID: 17668238).

In summary, despite the current thought that CUBN and AMN are only expressed by proximal tubular cells in the kidney, and that C-terminal mutations in *CUBN* resulting in non-progressive albuminuria only depend on failure to reabsorb albumin and this is not associated with loss of kidney function, there is also published evidence that is consistent with a further role of CUBAM in podocytes. We do not argue that this hypothesis is correct, just that it may be a potential interpretation of available evidence that merits further exploration based on our nephrocyte findings. Therefore, following the reviewer suggestions we discuss some of these published evidence in the revised versions of the manuscript. Determination of whether the proposed role of CUBAM in SD endocytosis may or may not be relevant for the mammalian kidney will require further investigation.

© 2021. Published by The Company of Biologists under the terms of the Creative Commons Attribution License (https://creativecommons.org/licenses/by/4.0/).

Minor points:

1. There is generally very little information on nephrocyte dissections in the methods. What are, for example, mature nephrocytes (line 157, p.6)?

Authors' response:
We thank the reviewer for pointing this out. We agree that "mature" nephrocytes does not mean much and it has been corrected in the revised manuscript. We also made an effort to expand the M&M section. Instead of referencing previous articles for the basic immunochemistry techniques, in the revised version we explicitly described them.

Reviewer #2

Major concerns and comments:

1. As the authors mentioned, recent studies have found that Cubulin and Amn are expressed in murine and human glomerular podocytes. It is reasonable to assume that the CUBAM-mediated endocytosis may also regulate slit diaphragm dynamics in mammalian podocytes, as it did in Drosophila nephrocytes. However, no consistent glomerular podocyte pathology has been found in human patients with Imerslund- Grasbeck syndrome or animal models of cubilin deficiency. This argues against a major role of CUBAM complex in regulating slit diaphragm dynamics in mammalian podocytes.

Authors' response:
We thank the reviewer for raising this interesting point regarding the human phenotype of *CUBN* mutations, a concern also manifested by reviewer 1. We agree with the reviewers that this phenotype has not been fully characterised. However, there is some recent information that may shed light into the potential contribution of podocyte CUBN to normal kidney function. In the first place we would like to consider the phenotypes we described in *Drosophila*, where mutations in CUBAM components result in lower density of SDs, but maintain still a fair number of diaphragms. Based on this, we would not expect a severe podocyte phenotype in humans if CUBAM played a similar role, but it could be possible that the phenotype become evident under stress conditions. Revising published data on Imerslund-Grasbeck syndrome (I-GS) patients we find that:

1. *CUBN* mutations may result in I-GS, in which the main phenotype is vitamin B12 deficiency, or in isolated proteinuria. Patients with isolated proteinuria have mutations affecting specific CUBN domains.
2. Currently, it is thought that patients with *CUBN* mutations resulting in isolated proteinuria have defects restricted to albumin reabsorption by proximal tubular cells. Indeed, Bedin et al., 2020 (PMID:31613795) recently described a large cohort of patients (n=39) presenting chronic isolated proteinuria and early childhood onset, associated with biallelic pathogenic CUBN variants. They concluded that "there is an important role for the C-terminal half of CUBN in renal albumin reabsorption and that albuminuria due to reduced cubilin function could be an unexpectedly common benign condition in humans that may not require any proteinuria-lowering treatment or renal biopsy".

While in principle we agree with this interpretation of the data, we argue against considering that they exclude a role of podocyte CUBN. In this regard, we would respectfully argue that science remains open to alternative interpretation of data once new knowledge becomes available, as it is the case for our data on CUBAM in *Drosophila*.
Thus, we would like the reviewers to consider the following arguments that potentially may open the door to further research along the lines identified by our *Drosophila* work, i.e., while not proving the clinical relevance of the *Drosophila* observation, they are at least consistent with such potential clinical relevance:

I. The Bedin cohort is mostly young, with a mean age at last follow-up of 10 to 26 years (table 1). This precludes conclusions on the long-term outcome of kidney function, as stated by the authors: "Although adverse long-term effects associated with *CUBN* deficiency cannot be fully excluded".
II. While Bedin figure 4.D shows that renal function of patients with biallelic filtered CUBN

© 2021. Published by The Company of Biologists under the terms of the Creative Commons Attribution License (https://creativecommons.org/licenses/by/4.0/).

variants (n = 35) was found to be declining according to normal aging, older patients are clearly underrepresented as compared to the comparator cohort. However, supplementary figure 1.D showing eGFR values taken at different disease progression times, discloses that the tree patients aged more than 35 had a dramatic decrease in eGFR over a short period of time.

There are multiple potential explanations for this observation. However, the data are also consistent with a SD recycling defect that may become relevant upon stress conditions, leading to rapid loss of kidney function. In this regard, CUBN variants associated with albuminuria had a greater impact on albuminuria in diabetic kidney disease, a condition in which albuminuria is associated with progressive loss of kidney function, than in controls (PMID: 30547231, PMID: 21355061). Furthermore, CUBN variants associated with proteinuria were also associated with renal function loss in two independent settings: ESRD in native kidneys and in transplanted kidneys (PMID: 22574174). This would not be predicted nor explained by an exclusive tubular role of cubilin in albuminuria.

III. They also say that “although not measured in all patients, the proportion of albumin in the urinary protein was higher than 50%, and urinary α1- or β2- microglobulin was mostly low or absent”. We agree with Bedin et al., 2020 that this argues against a generalized tubular defect in low molecular weight protein reabsorption and differs from other forms of tubular proteinuria, including megalin deficiency. However, these data may also be consistent with a mild increase in albumin (MW 66 KDa) filtration. And, as Bedin et al describe, this may not be deleterious as albuminuria has several adverse effects that depend on albumin uptake by tubular cells that will not take place in patients with mutations in *CUBN*. Then a relevant question now becomes, what about other larger proteins such as transferrin (MW 80 KDa), or IgG (MW 150 KDa) that are typically filtered in excess when the filtration barrier is not fully functional? Bedin et al do not provide data on this. However urinary protein was increased (mean 0.69 to 1.0 g/day), albumin accounted for around 60% of urinary protein and low molecular weight proteins were low or absent. Thus, other, likely larger, proteins contributed from mean 200 to mean 400 mg/day non-albumin proteins in urine in these patients. Therefore, to our knowledge this has not been explored in depth for patients carrying only-proteinuric CUBN variants. However, Wahlstedt-Fröberg et al (PMID: 12687456) analysed proteinuria in cubilin-deficient patients with selective vitamin B12 malabsorption. They reported that urinary transferrin was below the detection limit in controls and in 6/7 patients with mutations in *CUBN* with low proteinuria and low albuminuria, but was increased (up to 18-fold) in 6/6 patients with higher albuminuria. IgG was measured with a sensitive assay in only 2 controls (0.2mg/mmol creatinine) and 3 patients (0.3-0,8 mg/mmol, i.e., from 50% to 4-fold higher than in controls). In another study of proximal tubular function in I-GS, increased urinary excretion of transferrin was also reported (PMID: 24156255), and further reports also found increased urinary transferrin (1.17mg/mmol creatinine; normal<0.2) and IgG (0.98mg/mmol creatinine; normal<0.2) (PMID: 17668238). While it may be argued that these are also cubilin ligands, and their presence in urine may might be due to insufficient proximal tubular reabsorption, their larger size means that their presence in urine may also reflect and be compatible with higher filtration. Indeed, cubilin-independent transferrin tubular reabsorption was observed in mice (PMID: 20798259).

IV. The eGFR values reported by Bedin et al for their cohort were normal or even in the high normal range for younger patients (95% CI up to 172 ml/min/1,73m^2), but additionally four C-terminal missense CUBN variants were associated with albuminuria and an increased eGRF in population-based studies. Excessive filtration of albumin may result in higher eGFR as it may increase the oncotic pressure in the urinary side of the glomerular filtration barrier. Thus, these data may also be consistent with a mild increase in albumin filtration. Similarly, previous reports of I-GS patients also describe low serum creatinine values that, among other possibilities, may be consistent with glomerular hyperfiltration (PMID: 24156255), or confirmed hyperfiltration by increased creatinine clearance (PMID: 17668238).

In summary, despite the current thought that CUBN and AMN are only expressed by proximal tubular cells in the kidney, and that C-terminal mutations in *CUBN* resulting in non-progressive albuminuria only depend on failure to reabsorb albumin and this is not associated with loss of kidney function, there is also published evidence that is consistent with a further role of CUBAM in

© 2021. Published by The Company of Biologists under the terms of the Creative Commons Attribution License (https://creativecommons.org/licenses/by/4.0/).

podocytes. We do not argue that this hypothesis is correct, just that it may be a potential interpretation of available evidence that merits further exploration based on our nephrocyte findings. Therefore, following the reviewer suggestions we discuss some of these published evidence in the revised versions of the manuscript. Determination of whether the proposed role of CUBAM in SD endocytosis may or may not be relevant for the mammalian kidney will require further investigation.

2. The model in Figure 7 showed that slit diaphragm proteins are directly endocytosed by the CUBAM-mediated endocytosis. This speculation needs more evidence, since the authors did not show any interaction between slit diaphragm proteins and CUBAM complex, colocalization between these proteins, or other direct evidence.

Authors' response:
We agree with the reviewer that we do not present direct evidence showing that SD proteins are ligands for the endocytic CUBAM receptor. However, we do present indirect evidences.

When general endocytosis is blocked, for example in the clathrin knock-down experiments, we observe a strong accumulation of SDs in the plasma membrane, that folds and ingresses towards the interior of the cell. A similar accumulation of SD in internal membrane ingressions is also observed in CUBAM loss of function mutations. We speculate that these membrane ingressions result from a continuous addition of excess membrane that is inserted in the plasma membrane due to the high exocytic activity of nephrocytes, that is not compensated by endocytosis. Plasma membrane internalisation would drag externally located SDs towards internal positions, where they are never found in normal conditions, possibly because when this happens they are up- taken and degraded. The presence of these extra, internal SDs is consistent with a role of endocytosis, and of CUBAM in particular, in removing SDs from ectopic positions along the plasma membrane.

A stronger evidence for a direct involvement of CUBAM in SD turnover comes from our experiments showing that when membrane balance is restored in CUBAM mutants by ectopically expressing Mgl, ectopic SDs still accumulate in the plasma membrane at subcortical positions. This suggests that CUBAM can promote the correct turnover of SDs whereas Mgl cannot, indicating a possible direct interaction between CUBAM and SDs. Since SDs are very stable structures under normal circumstances (Carrasco-Rando et al. 2019), their interaction with CUBAM receptor must be an infrequent event hampering direct analyses of such association.

In conclusion, we believe that our data indicating that CUBAM is an endocytic receptor for SD proteins, albeit indirect, are solid enough to warrant this hypothesis to be included in the discussion section of the manuscript as well as in the model shown on Figure 7.

3. Some results in this paper (and from literatures) could be interpreted against CUBAM-mediated endocytosis of slit diaphragm proteins. For example, the phenotypes of knocking-down genes in the clathrin pathway and major components of endocytic pathway appear different from that of CUBAM mutants. The phenotype of chc and Rab5 knockdown (Figure 5D and 5E) exhibited clear differences in slit diaphragm localization from CUBAM mutants. If the endocytosis of slit diaphragm proteins were mainly mediated by CUBAM complex, shouldn't we see similar defects?

Authors' response:
Regarding the phenotypes of *Chc* and *Rab5* knockdowns shown in previous figures 5D and 5E, we want to thank the reviewer for helping us notice this apparent inconsistency in our data. Our data indicate that a substantial fraction of global endocytosis in nephrocytes is mediated by CUBAM. In particular, we see a strong reduction in the uptake of Idgf2 and/or dextran in *cubn-2* and *dAMN* mutants (Figures 1, S2, S6). However, we do not rule out that in the absence of CUBAM some endocytosis still occurs. In fact, although with very little frequency, some clathrin-coated structures are observed in mutants for CUBAM components. Thus, it would be expected that blocking general endocytosis components such as Clathrin or Rab5 would result in stronger endocytosis phenotypes than just blocking the CUBAM complex. In addition, both Chc and Rab5 have cellular functions beyond mediating endocytosis at the plasma membrane. Thus, phenotypes for these genes would be expected to be more pleiotropic than those of CUBAM.

© 2021. Published by The Company of Biologists under the terms of the Creative Commons Attribution License (https://creativecommons.org/licenses/by/4.0/).

We realise that using the strong drivers *sns-GAL4* and *pros-GAL4* to knockdown these global endocytic regulators, we obtained very strong SD intracellular aggregates that could be interpreted as qualitatively different from those of CUBAM mutants.
Considering the reasoning exposed above, we assumed that a milder attenuation of *Chc* or *Rab5*, using the weaker driver *cubn-2-GAL4*, might be sufficient to allow a mostly normal differentiation of the nephrocytes and still reveal a requirement of endocytosis in SDs distribution or turn-over. Accordingly, as shown in the new panels 5D and 5E, under these conditions we observed internal SDs aggregates as well as SDs at cortical positions, phenotypes equivalent to those of LOF mutants in CUBAM components.

In addition, please consider the phenotypes we show in Figure S5 for the endocytic mutant *dor*^{8}, lacking *Drosophila* CORVET and HOPS functions. They clearly display central SD aggregates, acentric nuclei as well as a small decrease in the number of cortical SDs together with some subcortical ectopic SDs, further supporting the idea that interference with early endocytosis induces similar phenotypes as CUBAM loss of function.

4. The results of partial rescue of slit diaphragm ingressions by silencing sec6 or sec15, overexpression of activated Rab5 or megalin in CUBAM mutant nephrocytes are confusing. These genetic manipulations all partially rescued slit diaphragm ingressions of CUBAM mutants, yet each showed different slit diaphragm mislocalizations on cell membrane. If the phenotypes in CUBAM mutants were due to imbalance between endocytosis and exocytosis, why only the ingressions were rescued? What is the reason for the phenotypic differences among these genetic manipulations?

Authors' response:
We suggest that re-establishing membrane balance rescues the big membrane ingressions found in mutants affecting CUBAM components without restoring the dynamism of SD proteins, which is still aberrant. This is the main reason supporting a direct role of CUBAM and not of general endocytosis in SDs degradation and/or recycling. The SD phenotypes in CUBAM mutants after expression of Mgl, Rab5 or sec15-RNAi are qualitatively similar. We detect rescue of membrane and SD large ingressions, a lower density of SDs at the nephrocyte surface and finger-like protrusions containing SDs components at the periphery (figure 6G). However, we realise that the overexpression of *Rab5*^{Q88L}, shown in previous panel 6A, displays a dotted distribution of Duf that is not observed in the other genetic manipulations. We have repeated this experiment overexpressing *UAS-Rab5* instead of the activated form *UAS-Rab5*^{Q88L}. With *UAS-Rab5* we still observe rescue of membrane and SDs ingressions and interestingly, it is also evident that Rab5 induces partial internalization of Duf but not of Pyd, explaining the dotted pattern that was more dramatic with *UAS-Rab5*^{Q88L}. Thus, in the revised version of the manuscript we use Rab5 overexpression instead of its activated form (Figure 6A). The new panel shows lower density of SD at the nephrocyte surface and finger-like protrusions from the plasma membrane labelled with Duf and Pyd, together with the absence of large ingressions of SDs. It is also evident some Duf internalization induced by Rab5 overexpression that is not relevant for the conclusions of the experiment.
We thank the reviewer for helping us notice that some of the images and experimental manipulations we selected for the manuscript figures were not optimal.

5. In Panel A of the model, the authors hypothesize that CUBAM facilitates the SD components degradation or recycling through a cortical tubular network. However, there is no direct evidence to demonstrate this. To claim that "the main function of the nephrocyte cortical tubular network is to quick recycling of receptor- ligand complexes", more evidence is needed. Maybe the authors could consider genetically alter the nephrocyte cortical tubular network and examine its effect on the recycling of receptor-ligand complexes.

Authors' response:
We observed in our TEM images as well as in our 3D FIB-SEM models the presence of a conspicuous cortical tubular network adjacent to the plasma membrane in the wild-type (see Supplementary file S1) that was virtually absent in *cubn-2* mutants. This suggests that the cortical tubular network is an early endocytic pathway organelle in nephrocytes. Interestingly, a morphologically similar

© 2021. Published by The Company of Biologists under the terms of the Creative Commons Attribution License (https://creativecommons.org/licenses/by/4.0/).

apical tubular network is present in proximal tubular cells in mammals, which also express the CUBAM complex and are highly endocytic. This tubular network was shown to be involved in membrane recycling from endocytic vacuoles to the cell membrane, being a type of sorting endosome compartment (Hatae et al 1996). This suggests a similar function in nephrocytes. However, we agree with the reviewer that there is no evidence of a direct role of the cortical tubular network in SD degradation or recycling, other than the general concept that endocytosed receptor- ligand complexes are targeted to sorting endosomes for their sorting into the different recycling or degradation routes they take. Thus, we have opted for substantially change our discussion about the tubular network in nephrocytes to emphasise their probable role in rapid recycling of CUBAM and possibly other receptor complexes, back to the plasma membrane, as well as contributing to membrane homeostasis. Their possible specific role in the sorting, degradation or recycling of SDs is now just implied as a general function expected from the sorting endosome compartment.

6. In Panel B of the model, it is confusing why "a reduction in the recycling of SD components" can lead to accumulation of extra SDs at subapical locations and in deep regions of the enlarged labyrinthine channels. If SD recycling is reduced as shown in the model, shouldn't the number of SD be reduced as well? If extra SD were found in CUBAM mutants, wouldn't it suggest an increased SD recycling or an increased production of new SD proteins?

<u>Authors' response</u>:
We thank the reviewer for noticing this mistake. The text should read: "This effect, combined with a reduction in CUBAM-mediated endocytosis of damaged SD components, can lead to the observed accumulation of extra SDs at subapical locations and in deep regions of the enlarged labyrinthine channels". We changed the revised version of the manuscript accordingly and apologise for the misunderstandings it caused. The second part of the referee observation raises an interesting issue. Accumulation of SDs in nephrocytes can be caused not only by reduced uptake and degradation but also by increased synthesis of SD components. However, we do not favour this hypothesis. Data from our lab indicates that SDs are very stable structures under normal conditions and that SD remodelling is mainly regulated at the post-transcriptional level, for example by phosphorylation (Tutor et al. 2014). Currently, there is no evidence that modulating endocytosis would have a coordinated effect on the transcription of the numerous proteins that constitute a SD complex.

7. The discussion for the model is confusing. In Page 19, line 510, it says "possibly by promoting the endocytosis of SD components for its posterior degradation and/or recycling (Fig. 7)". What does "posterior degradation and/or recycling" mean?

<u>Authors' response</u>:
Our hypothesis suggests that SD components are removed from the plasma membrane by CUBAM-mediated endocytosis. However, we realise that we should have made it clearer in our model that CUBAM probably participates in the removal of damaged components or SD proteins that had been targeted for degradation, for example by phosphorylation events. We do not suggest that SDs are constantly being endocytosed and recycled back to the membrane. We have extensively changed the discussion of our model to better reflect this hypothesis.

Reviewer #3

<u>*Major comments*</u>:

1. I don't think that the claim of Idgf2 being an endogenous ligand for this endocytic receptor complex (line 155) is properly supported by the data, as the effect could also be indirect. Please tone down this statement if you can't provide e.g. biochemical binding data.

<u>Authors' response</u>:
We agree with the reviewer that in order to demonstrate that Idgf2 is a CUBAM ligand we would need direct physical interaction data, that we do not have. Thus, in the revised version we toned down this statement.

2. The conclusion of decreased endocytosis in mutants is based on Idgf2-GFP, dextran

© 2021. Published by The Company of Biologists under the terms of the Creative Commons Attribution License (https://creativecommons.org/licenses/by/4.0/).

uptake and TEM experiments, the latter without quantification. It would be useful to expand these data by visualizing and quantifying clathrin, Rab5 and Rab7 structures in different genotypes using confocal microscopy.

Authors' response:
In the revised version of the manuscript, we quantitate the number of clathrin-coated structures in TEM micrographs from wild-type and *cubn-2*$^{E3\text{-}1}$ mutants, showing a dramatic reduction of these structures in the mutant. In addition, we included a new Supplementary Figure showing that in *dAMN*^{3} nephrocytes dextran uptake is extremely compromised compared to the wild-type. However, we agree with the reviewer that showing an effect on early Rab5 endosomes could provide additional evidence in this direction. Unfortunately, the Rab5 antibody that is available to us, as well as the Rab5- YFP reporter line we tested have high background and are not suitable for quantification. We do have data regarding clathrin distribution in the wild-type and CUBAM mutants. Clathrin is exclusively and uniformly detected in the cortical region of wild-type nephrocytes and it does not form vesicles recognizable at the confocal resolution. In this regard is similar to Cubn or Cubn-2 distributions. In *cubn-2*$^{E3\text{-}1}$ mutants, clathrin is similarly localized at the cortex. However, since in this genetic background the labyrinthine channels expand to more central locations, clathrin is also detected surrounding these expanded channels. Since chathrin cannot be resolved to form vesicles in either genotype, we believe it is not a suitable marker to examine endocytosis.
Despite the previous observations, we believe the data we present in the revised version of the manuscript showing reduced endocytosis in CUBAM mutants is already undeniable.

Minor comments:

3. Representative images should be included for Fig 4i (e.g. as supplemental material)

Authors' response:
We agree with the reviewer and have included them in the revised version of panel 4I.

4. Do Fig 6h-j also show tannic acid impregnation TEM? Why are invaginations colored light blue in panel i? Why are black arrowheads pointing to the plasma membrane in panel j? Please clarify these.

Authors' response:

We are sorry for the confusion in Figure 6 legend. In the revised version of the manuscript we chose to remove the TEM data from figure 6. In any case, Figure 6H-J did not show tannic acid impregnation micrographs, but regular TEM.

Second decision letter

MS ID#: DEVELOP/2021/199894

MS TITLE: Endocytosis mediated by an atypical CUBAM complex modulates slit diaphragm dynamics in nephrocytes.

AUTHORS: Alexandra Atienza-Manuel, Vicente Castillo-Mancho, Stefano De Renzis, Joaquim Culi, and Mar Ruiz-Gómez
ARTICLE TYPE: Research Article

I am happy to tell you that your manuscript has been accepted for publication in Development, pending our standard ethics checks. I reviewed this revision myself and find the new version thoroughly addresses all of the concerns raised by the original reviewers very well. Thanks for submitting this really nice work to Development.

© 2021. Published by The Company of Biologists under the terms of the Creative Commons Attribution License (https://creativecommons.org/licenses/by/4.0/).
